# Multi-omics analysis reveals neutrophil heterogeneity and key molecular drivers in sepsis-associated acute kidney injury

**DOI:** 10.3389/fimmu.2025.1637692

**Published:** 2025-10-02

**Authors:** Tianle Cheng, Yong Xu, Ziwei Liu, Yanchen Wang, Ze Zhang, Wenlin Huang

**Affiliations:** ^1^ Department of Urology, Zhuzhou Hospital Affiliated to Xiangya School of Medicine, Central South University, Zhuzhou, Hunan, China; ^2^ Pharmacy Department, Zhuzhou Hospital Affiliated to Xiangya School of Medicine, Central South University, Zhuzhou, Hunan, China; ^3^ Department of Urology, The Third People′s Hospital of Datong, Datong, Shanxi, China

**Keywords:** sepsis, acute kidney injury, neutrophil heterogeneity, neutrophil extracellular traps (NETs), multi-omics, peptidyl arginine deiminase 4, biomarkers

## Abstract

Sepsis-associated acute kidney injury is a critical condition driven by immune dysregulation, particularly involving neutrophils, yet their heterogeneity and molecular contributions remain underexplored. This study employed a multi-omics approach, integrating single-cell and bulk RNA sequencing from 21 sepsis samples and Escherichia coli-induced sepsis datasets, alongside bioinformatics, machine learning, and experimental validation in a rat model and human peripheral blood. We identified four neutrophil subtypes—pro-inflammatory, anti-inflammatory, mature, and immature—revealing a significant increase in pro-inflammatory neutrophils in sepsis (40.53% versus 4.19% in controls) and a decrease in anti-inflammatory neutrophils (18.43% versus 27.04%). Four hub genes, peptidyl arginine deiminase 4, caspase 4, complement receptor 1, and mitogen-activated protein kinase 14, were pinpointed as key drivers, with peptidyl arginine deiminase 4 mediating neutrophil extracellular trap formation and exacerbating renal damage. In a rat model, peptidyl arginine deiminase 4 knockdown reduced trap formation and alleviated kidney injury (p-value less than 0.01). Human samples confirmed elevated gene expression in sepsis (p-value less than 0.05). These findings highlight neutrophil heterogeneity and molecular mechanisms in sepsis, with potential implications for sepsis-associated acute kidney injury (SAKI), proposing novel biomarkers and therapeutic targets for precision medicine.

## 1 Introduction

Sepsis, characterized by life-threatening organ dysfunction due to a dysregulated host response to infection, presents a complex pathophysiology with heterogeneous onset and prognosis, prompting extensive research (1). Acute kidney injury is the most frequent organ damage in sepsis, particularly in intensive care settings, with an incidence reaching 57.3 (2). In urosepsis, kidney damage occurs through multiple pathways: bacterial toxins such as lipopolysaccharides directly impair renal tubular epithelial and endothelial cells, systemic inflammation disrupts hemodynamic stability leading to reduced renal perfusion (3), and local bacterial effects within the urinary tract exacerbate injury (4–6). This interplay creates a vicious cycle, where urosepsis triggers acute kidney injury, and worsening kidney function intensifies systemic inflammation and toxin accumulation (5, 6). Consequently, early infection control is critical to reduce renal burden, while preserved kidney function is vital for successful sepsis management (2, 6). Clinically, timely relief of urinary tract obstruction in urosepsis can effectively reverse systemic inflammation and kidney damage.

Neutrophils, the most abundant white blood cells, are pivotal in the early immune response to sepsis, serving as a routine marker for assessing inflammation severity and prognosis (7). However, neutrophils are a heterogeneous population, with distinct subtypes emerging during granulopoiesis and exhibiting diverse roles across tissues and developmental stages (7). Recent studies have identified various neutrophil subpopulations, highlighting their divergent functions in inflammation (8, 9). Despite these advances, the specific roles of neutrophil heterogeneity in sepsis-associated acute kidney injury, particularly in urosepsis, remain underexplored. Single-cell transcriptomics offers a powerful approach to dissect this heterogeneity, uncover novel biomarkers, and elucidate immune interactions, yet its application in sepsis-related kidney injury is limited.

Given the lack of systematic studies on neutrophil heterogeneity and molecular mechanisms in sepsis-associated acute kidney injury, this study was necessary to address this gap. We aimed to characterize neutrophil subtypes and identify key molecular drivers in sepsis-associated kidney injury, focusing on Escherichia coli-induced sepsis. Notably, while the datasets used in this study represent general sepsis cohorts without explicit SAKI diagnostic criteria (e.g., KDIGO stages based on serum creatinine or urine output), our findings on neutrophil heterogeneity provide insights into sepsis-induced immune dysregulation that may contribute to SAKI. By integrating single-cell and bulk RNA sequencing with experimental validation, we sought to uncover novel biomarkers and therapeutic targets, contributing to precision medicine strategies for sepsis management and improving outcomes in patients with sepsis-associated acute kidney injury.

## 2 Materials and methods

### 2.1 Single-cell data collection

Two single-cell transcriptome datasets related to sepsis were obtained from the Gene Expression Omnibus (GEO) database (https://www.ncbi.nlm.nih.gov), specifically GSE175453 (which includes 9 samples) and GSE167363 (which includes 12 samples). In total, 21 single-cell transcriptomic samples were utilized for various analyses, including cell annotation, identification of neutrophils and their subtypes, pseudo-time series analysis, and the identification of differentially expressed genes in neutrophils from healthy individuals compared to those from sepsis patients. These datasets, capturing peripheral blood mononuclear cells (PBMCs) and leukocytes from sepsis patients, reflect systemic immune responses potentially relevant to organ dysfunction, including SAKI, though they lack explicit kidney-specific clinical annotations based on KDIGO criteria (e.g., serum creatinine or urine output).

### 2.2 Data quality control and batch removal

Cell tags, genes, and expression matrices were obtained directly from the GEO database, and the Seurat v4 package (10) was utilized to process the data. The filtering criteria applied to the pooled samples included: 300 < nFeature_RNA < 2000, nCount_RNA < 40000, percent.mt (ratio of mitochondrial genes) < 30, and log10GenesPerUMI > 0.85.

### 2.3 Dimensionality reduction, annotation, differential expression

The Seurat pipeline was utilized to reduce the dimensionality of the combined cell data. First, principal component analysis (PCA) was conducted and visualized, followed by the creation of an elbow plot to identify the most significant principal components. The harmony algorithm (11) was then employed to address batch effects among various samples. After that, Dimensionality reduction was performed on the selected principal components (PCs) using both UMAP and t-SNE. Subsequently, cluster plots were generated to determine the optimal resolution parameter for partitioning cells into biologically distinct clusters.

The dataset was annotated into different cell types, such as T lymphocytes, B lymphocytes, neutrophils, and plasma cells, using the CellMarker2 database. Visualization was enhanced through bubble plots of marker genes and UMAP annotation plots.

Differential expression analysis was carried out using the FindAllMarkers function, aiming to identify differentially expressed genes within the same group across different clusters, as well as those within the same cluster but across different groups. Genes were selected as differentially expressed if they met the criteria of |logFC| > 0.25 and a P-value < 0.05.

### 2.4 Pseudo-time analysis

Pseudo-time trajectory analysis was conducted using the Monocle package (12). Highly variable genes were selected based on an average expression threshold (>0.1) and ranked within the top 2000 by coefficient of variation. Dimensionality reduction was performed using the DDRTree algorithm (13), and cellular trajectories were visualized through scatter plots generated by the built-in plot_cell_trajectory function. Genes were partitioned into two distinct clusters based on their pseudo-temporal expression patterns. Differential gene expression along the pseudo-time axis was visualized using the plot_pseudotime_heatmap function. Gene Ontology (GO) enrichment analysis for temporally dynamic pathways was subsequently performed with the clusterProfiler package (14), employing a significance threshold of *p* < 0.05 and false discovery rate (FDR) < 0.25.

### 2.5 Cell-cell communication analysis

Cell-cell communication analysis was performed on annotated cell subpopulations using the CellChat package (15). Ligand-receptor interactions were predicted based on a predefined signaling pathway database. Key communication patterns were visualized through three types of diagrams: (1) Cell-cell interaction networks representing connections between cell clusters; (2) Incoming signaling heatmaps showing receptor activity in target cell types; (3) Outgoing signaling heatmaps displaying ligand expression in source cell types. All visualizations were generated using default settings of the CellChat package.

### 2.6 Metabolic pathway analysis

Metabolic pathway activities across cell types were analyzed using the scMetabolism package (16). Heatmaps were generated to visualize pathway activity differences.

### 2.7 Bulk RNA-seq data collection

Publicly available datasets were retrieved from the Gene Expression Omnibus (GEO) database The following datasets were included:

1. Escherichia coli-induced sepsis dataset (GSE237960): Due to the scarcity of urosepsis-specific datasets in public repositories, this dataset was selected as a surrogate based on the clinical relevance of *E. coli* as the predominant pathogen in urinary tract infections. The GSE237960 dataset was selected as a surrogate for urosepsis, given that E. coli is the predominant pathogen in urosepsis (70-80% of cases) and shares immune mechanisms such as neutrophil activation, NET formation, and cytokine responses with general E. coli sepsis (17–19). Whole blood RNAseq data of 4 patients with Escherichia coli sepsis (experimental group) and 4 normal people (Control group) were included; Platform: Affymetrix Human Genome U133 Plus 2.0 Array; Purpose: Identification of differentially expressed genes (DEGs) and exploratory data mining.
2. Sepsis training/validation dataset (GSE185263): Platform: Affymetrix Human Gene 1.0 ST Array (GPL6244); Purpose: Construction of a support vector machine (SVM) model, immune cell infiltration analysis, and preliminary validation of candidate genes.
3. External validation dataset (GSE243217): Platform: Affymetrix Human Gene 1.0 ST Array (GPL6244); Purpose: Independent validation of diagnostic performance for hub genes.
4. NETF pathway gene set: A curated list of 132 genes associated with neutrophil extracellular traps (NETs) formation, a process where neutrophils release chromatin fibers to immobilize pathogens during infection or inflammation. The data source is the KEGG database (https://www.kegg.jp/, accession: hsa04613).

### 2.8 Bulk RNA-seq data preprocessing

Raw transcriptomic data were downloaded from the GEO database using the GEOquery package (20). Platform-specific annotation files were utilized to map probes to gene symbols. Expression matrices were normalized via the RMA algorithm (21), including background correction, quantile normalization, and probe summarization. For genes with multiple probes, expression values were averaged to generate gene-level measurements.

### 2.9 Differential expression analysis

Differentially expressed genes (DEGs) were identified in the *E. coli*-induced sepsis dataset using the limma package (21). Linear models comparing sepsis patients and controls were fitted after variance-stabilizing normalization. Statistical significance was defined as |log_2_FC| > 0.5 and *p* < 0.05, with FDR correction applied to control false discoveries (FDR < 0.1).

### 2.10 Functional enrichment analysis (GO/KEGG)

GO and KEGG analyses were performed using the clusterProfiler package (14, 22) Gene sets included pseudotime-associated DEGs (Section 2.4) and *E. coli*-sepsis DEGs (Section 2.8). Significant terms (FDR < 0.05) were visualized as lollipop charts (top 9 KEGG pathways) and dot plots (top 3 GO terms per category).

### 2.11 Venn diagram, machine learning (SVM), and single-gene ROC analysis

The CNSKnowall Cloud Platform (https://www.cnsknowall.com/veen) was utilized to generate a Venn diagram illustrating the overlap between NETF genes, neutrophil differential genes, and differential genes identified through Bulk-RNAseq. This analysis enabled the identification of neutrophil-urosepsis differential genes. A linear SVM model was trained on 70% of GSE185263 and validated on 30%, with diagnostic performance assessed by ROC curves using the pROC package (23). Hub genes with AUC > 0.7 were prioritized.

### 2.12 Immune infiltration analysis

To characterize the immune microenvironment in urosepsis, samples from the GSE185263 dataset were stratified into normal and sepsis groups. Immune cell infiltration profiles were quantified using the xCell algorithm (24), which estimates the abundance of 64 immune and stromal cell types based on gene expression signatures. The proportions of infiltrating immune cells were visualized through stacked bar plots generated with the ggplot2 package (version 3.4.0).

To identify differential immune infiltration between high-risk and low-risk subgroups (defined by the hub gene expression signature), the Wilcoxon rank-sum test was applied with a significance threshold of *p* < 0.05. Statistically significant cell types were further visualized using violin plots to display distribution differences. Associations between hub genes (PADI4, CR1, MAPK14, CASP4) and immune cell infiltration were analyzed via Spearman correlation and represented as lollipop plots, where the dot size and color intensity reflected correlation coefficients (*ρ*) and significance levels, respectively.

Additionally, the interplay between hub genes and immune checkpoint molecules (e.g., PD-L1, TIM-3, CTLA-4) was explored through heatmaps, with hierarchical clustering applied to both rows (genes) and columns (checkpoint genes). Immune cell types showing significant co-enrichment patterns were highlighted in enrichment bubble plots, where bubble size indicated the -log10(*p*-value) and color denoted enrichment scores.

### 2.13 Gene set enrichment analysis of hub genes

To investigate the biological pathways associated with hub genes in the disease, Gene Set Enrichment Analysis (GSEA) was performed using the training dataset from GSE185263. The “c2.cp.kegg.v7.0.symbols.gmt” gene set from the Molecular Signatures Database (MSigDB) was used as the reference gene set. First, Spearman correlation coefficients between each hub gene and all other genes in the dataset were calculated using the R package “psych” (version 2.5.3) (25). For each hub gene, a ranked gene list was generated by sorting all genes in descending order based on their correlation coefficients. Subsequently, GSEA was conducted using the R package “clusterProfiler” (version 3.8) to explore the potential functional roles of the hub genes (14). Analysis parameters were set at p < 0.05, FDR < 0.25, and |NES| > 1, with hub gene enrichment results visualized and reported.

### 2.14 Consensus clustering analysis

In this study, data preprocessing and consensus clustering were performed to identify the optimal clustering approach. Initially, a specific gene set, denoted as gene_Consensus, was selected from the raw dataset. Each gene (row) was then subjected to median centering by subtracting its row-specific median. For the consensus clustering analysis, Pearson correlation was employed as the distance metric, with partitioning around medoids (PAM) as the clustering algorithm. The analysis parameters were set as follows: a maximum cluster number of 6, 1000 iterations, a subsampling rate of 80% of the samples, inclusion of all features, use of complete observations for correlation calculations, and a fixed random seed of 814 to ensure reproducibility. The optimal number of clusters (k) was determined by incrementally increasing k from 2 to 6, with k = 2 identified as the optimal clustering solution based on clustering stability.

### 2.15 Subcellular localization analysis

To investigate the subcellular distribution of hub genes, subcellular localization analysis was performed using the GeneCards database (https://www.genecards.org/). The localization of these genes to specific organelles, along with their confidence scores, was visualized as bar plots using the R package “ggplot2”.

### 2.16 DGIdb drug interaction analysis

To explore existing drugs associated with the identified hub genes, drug-gene interaction data were retrieved from the DGIdb database (https://www.dgidb.org/). The relationships between hub genes and related drugs, including their interaction scores, were visualized as bar plots using the R package “ggplot2”.

### 2.17 Establishment of the sepsis rat’s model

The experiment utilized male Wistar rats (8–10 weeks old, 160–200 g; SLAC Laboratory Animal Co., Ltd., Shanghai, China), housed at 22 °C with 50% humidity and a 12:12 h light–dark cycle, with ad libitum access to standard chow and water. Rats were acclimated to the environment for at least one week prior to experimentation. All procedures were approved by the Institutional Animal Care and Use Committee (IACUC) and complied with national and international guidelines for animal experimentation. Rats were randomly assigned to three groups (n=3 per group): Sham group (laparotomy without cecal ligation or puncture), CLP group (cecal ligation and double puncture with a 20-gauge needle to induce sepsis), and CLP + sh-PADI4 group (CLP surgery followed by tail vein injection of 1×10^9 TU PADI4-targeting shRNA adenovirus). The CLP model was performed under aseptic conditions. Rats were anesthetized with 2.5% isoflurane, underwent midline laparotomy, and the cecum was ligated below the ileocecal valve and punctured twice with a 20-gauge needle, with a small amount of fecal matter extruded before abdominal closure. The Sham group underwent cecal exposure without ligation or puncture. Adenovirus was administered via slow tail vein injection, with all interventions conducted under anesthesia. Rats were fasted for 8–12 hours pre-surgery, recovered in a warm environment post-surgery, and monitored closely, adhering to predefined humane endpoints to minimize suffering. At 48 hours post-CLP, rats were euthanized under deep anesthesia, blood was collected via cardiac puncture, and serum was stored at -80°C. Both kidneys were harvested—one fixed in 10% neutral-buffered formalin or 4% paraformaldehyde for histological and immunofluorescence analysis, the other snap-frozen in liquid nitrogen and stored at -80°C for molecular assays. Aseptic techniques were maintained throughout, and sepsis-related tissues were disposed of according to biosafety protocols. The animal study was approved by the Institutional Animal Care and Use Committee of The Second Xiangya Hospital, Central South University (Approval No: 2022711).

### 2.18 Western blotting analysis

Kidney tissues from the CLP and Sham groups were homogenized in RIPA buffer containing protease inhibitors and centrifuged at 12,000 × g for 15 min at 4°C to extract proteins. Protein concentrations were determined using the BCA method. Thirty micrograms of protein were mixed with loading buffer, heated at 95°C for 5 min, separated by 10% SDS-PAGE, and transferred to a PVDF membrane. The membrane was blocked with 5% non-fat milk for 1 h, incubated overnight at 4°C with primary antibodies against PADI4 (1:1000, Abcam) and β-actin (1:5000, Sigma-Aldrich), washed with TBST, and incubated with HRP-conjugated secondary antibodies (anti-rabbit 1:5000, anti-mouse 1:10000) for 1h. After further TBST washes, bands were visualized using ECL detection. Band intensities were quantified with ImageJ, and PADI4 expression was normalized to β-actin.

### 2.19 Immunofluorescence staining

Kidney tissues from experimental groups were fixed in 4% paraformaldehyde at 4°C for 24–48 h, dehydrated, and embedded in paraffin. Sections (4–5 µm) were cut, mounted on adhesive slides, and baked at 60°C for 30 min. After deparaffinization in xylene and rehydration through a graded ethanol series, antigen retrieval was performed in citrate buffer (pH 6.0) at 95°C for 15 min. Sections were cooled, washed with PBS, and blocked with 5% goat serum in PBS for 30 min at room temperature. Sections were then incubated with anti-citH3 antibody (1:200, Abcam) overnight at 4°C, washed with PBS, and incubated with Alexa Fluor 488-conjugated anti-rabbit secondary antibody (1:500, Thermo Fisher Scientific) for 1 h at room temperature in the dark. Nuclei were counterstained with DAPI for 5 min, and slides were mounted with anti-fade medium. Images were captured using a fluorescence microscope and quantified for citH3-positive cells using ImageJ software.

### 2.20 Hematoxylin and eosin staining

Kidney tissues from experimental groups were fixed in 10% neutral-buffered formalin at room temperature for 24–48 h, dehydrated through a graded ethanol series, cleared in xylene, and embedded in paraffin. Sections (4–5 µm) were cut using a microtome, mounted on adhesive slides, and baked at 60°C for 30 min. Sections were deparaffinized in xylene, rehydrated through decreasing ethanol concentrations, and stained with hematoxylin for 5 min. After rinsing in tap water, sections were differentiated in 1% acid alcohol, blued in ammonia water, and counterstained with eosin for 1 min. Sections were dehydrated, cleared in xylene, and mounted with a coverslip using mounting medium. Images were captured using a bright-field microscope, and histopathological changes were evaluated.

### 2.21 Quantitative real-time PCR analysis

Peripheral blood samples were collected from 5 sepsis patients and 5 healthy controls. And the study of these samples was approved by the Ethics Committee of Zhuzhou Hospital Affiliated to Xiangya School of Medicine, Central South University (Approval No: 2020175-01). Total RNA was extracted from whole blood using TRIzol reagent (Invitrogen, USA) per the manufacturer’s instructions. cDNA was synthesized from 1 µg total RNA using the PrimeScript RT reagent kit (Takara, Japan). Quantitative real-time PCR was performed on a CFX96 system (Bio-Rad, USA) with SYBR Green premix (Takara, Japan). Expression of PADI4, CASP4, CR1, and MAPK14 was measured, normalized to β-actin (primer sequences in **Table 1**). Relative gene expression was calculated using the 2^−ΔΔCt method.

**Table 1:** Primer sequences for qRT-PCR.

Gene	Forward primer	Reverse primer
PADI4	CAGGGGACATTGATCCGTGTG	GGGAGGCGTTGATGCTGAA
CASP4	TCTGCGGAACTGTGCATGATG	TGTGTGATGAAGATAGAGCCCAT
CR1	CACGAAGCCGCCAATTTGTC	CCCACTTGATCGTCATTGCTG
MAPK14	TCAGTCCATCATTCATGCGAAA	AACGTCCAACAGACCAATCAC
β-actin	CATGTACGTTGCTATCCAGGC	CTCCTTAATGTCACGCACGAT

### 2.22 Statistical analysis

All analyses were executed utilizing R programming language (v 4.2.2). The Wilcoxon test was harnessed to contrast differences, employing a statistical threshold of p < 0.05.

^1^ Material and Methods section can be placed in any of the following ways.

## 3 Results

### 3.1 Data filtering and cell population identification

**Figure 1:**
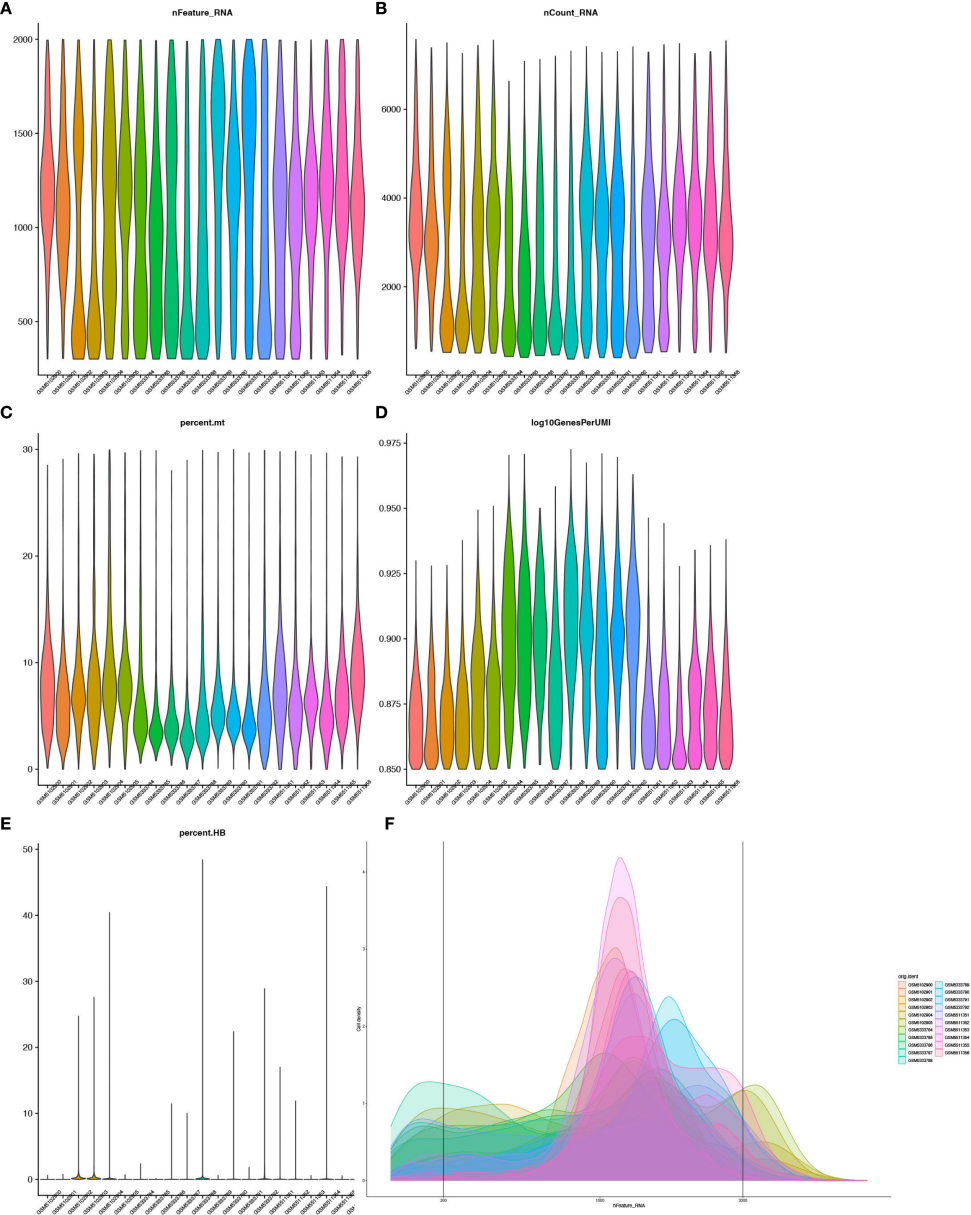
Quality control of single-cell RNA-seq data. **(A)** Violin plot showing gene detection per cell. **(B)** Total RNA counts per cell. **(C)** Mitochondrial gene percentage. **(D)** Gene detection efficiency (log10GenesPerUMI, 0.90-0.925). **(E)** Violin plot showing distribution of Hemoglobin Gene expression Proportion (percent.HB). **(F)** Density Distribution of gene detection across 21 single-cell RNA sequencing samples.

**Figure 2:**
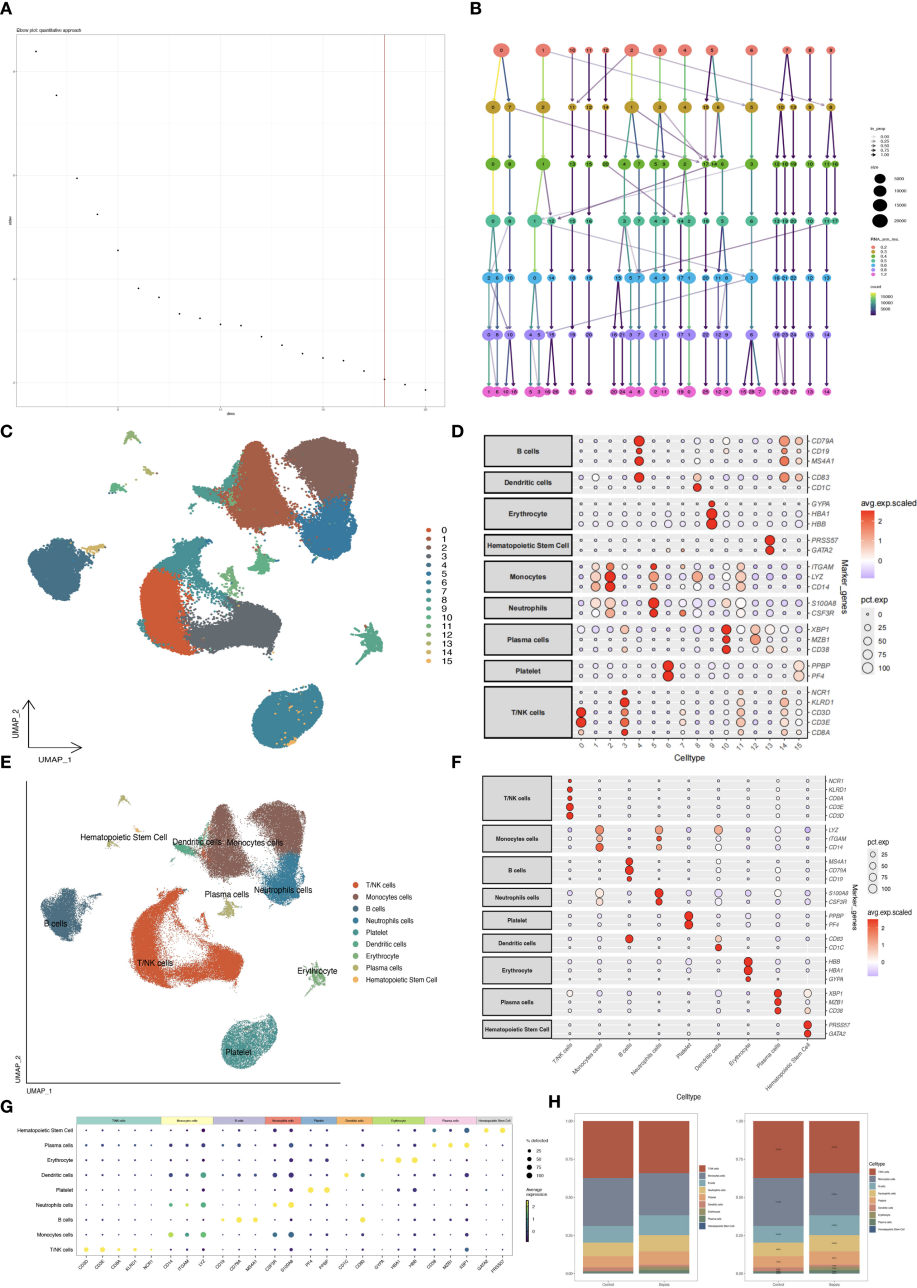
Single-cell dimensionality reduction and annotation. **(A)** PCA elbow plot selecting principal components. **(B)** Clustertree plot determining clustering resolution. **(C)** UMAP plot of 16 cell clusters. **(D)** Bubble plot of marker gene expression by cluster. **(E)** UMAP with annotated cell types. **(F, G)** Bubble plots of marker genes by cell type. **(H)** Bar plot comparing cell type proportions.

21 single-cell transcriptome samples were retrieved from the GEO database (GSE175453, 9 samples; GSE167363, 12 samples). Quality control was performed using Seurat v4 with the following criteria: 300 < nFeature_RNA < 2000, nCount_RNA < 40000, percent.mt < 30, and log10GenesPerUMI > 0.85. This confirmed a high-quality dataset (**Figures 1A–F**). Batch effects were corrected using the Harmony algorithm, followed by principal component analysis (PCA) to select significant components (**Figure 2A**). UMAP dimensionality reduction was applied with an optimal resolution determined by cluster tree analysis (**Figure 2B**), clustering cells into 16 groups with distinct marker gene expression profiles (**Figure 2D**). These were annotated as 9 distinct cell types, including B cells, T/NK cells, monocytes, and neutrophils (**Figures 2C, E**). In the sepsis group, the proportion of neutrophils was significantly elevated (10.63% vs. 8.94% in the healthy group), while T/NK cells showed a slight reduction (34% *vs*. 37%), and monocytes remained consistent (27%, **Figure 2H**). Marker gene analysis validated cell identities, with neutrophils highly expressing S100A8 and CSF3R, and T/NK cells expressing CD3E and KLRD1 (**Figures 2F, G**), providing a foundation for subsequent neutrophil-focused analyses.

### 3.2 Neutrophil subtype classification

**Figure 3:**
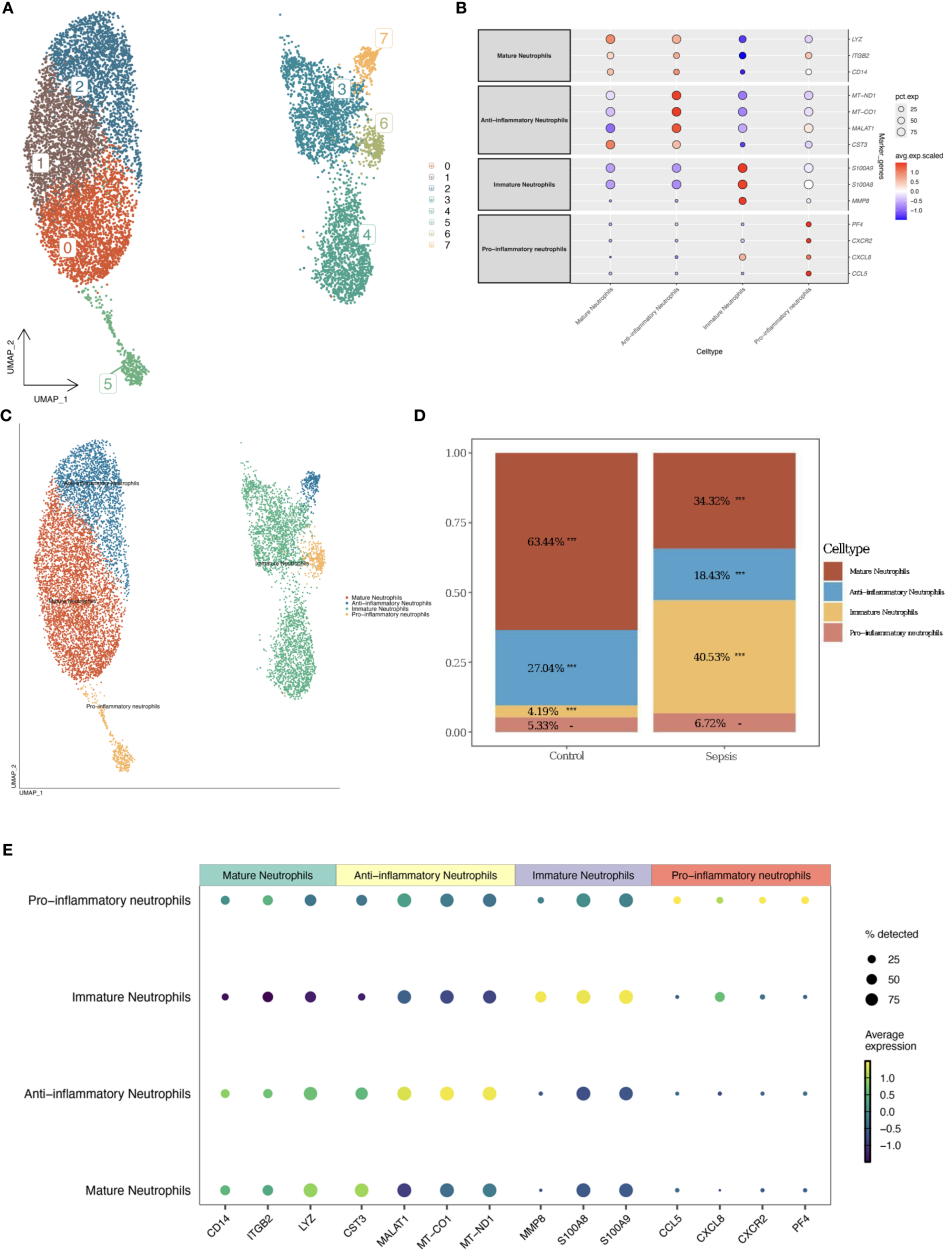
Neutrophil subtype classification. **(A)** UMAP plot of neutrophil clusters. **(B)** Bubble plot of marker gene expression by subtype. **(C)** UMAP with annotated subtypes (mature, immature, pro-inflammatory, anti-inflammatory). **(D)** Bar plot comparing subtype proportions. **(E)** Enhanced Marker Gene Bubble Plot (Cell Types).

Neutrophils were subdivided into 8 clusters using UMAP, which were further categorized into four subtypes based on immune-related and metabolic gene expression profiles: mature neutrophils, immature neutrophils, anti-inflammatory neutrophils, and pro-inflammatory neutrophils (**Figures 3A, C**). Anti-inflammatory neutrophils, defined by elevated MT-ND1, MT-CO1, MALAT1, and CST3, were more prevalent in the control group (27.04% *vs*. 18.43% in sepsis, **Figure 3D**), while pro-inflammatory neutrophils, distinguished by PF4, CXCR2, CXCL8, and CCL5, exhibited a slight increase in sepsis (6.72% *vs*. 5.33% in controls, **Figure 3D**), suggesting an intensified inflammatory state. Anti-inflammatory neutrophils were identified by high expression of MT-ND1, MT-CO1, MALAT1, and CST3, which are associated with mitochondrial homeostasis and inflammation resolution (26–28). Pro-inflammatory neutrophils were characterized by elevated PF4, CXCR2, CXCL8, and CCL5, promoting chemotaxis and inflammatory amplification in sepsis (29–33). Minimal cross-expression of marker genes across subtypes validated the robustness of this classification (**Figures 3B, E**). The substantial increase in immature neutrophils in the sepsis group (40.53% *vs*. 4.19% in controls, **Figure 3D**) underscored their mobilization during acute inflammation. The decrease in anti-inflammatory neutrophils (18.43% *vs*. 27.04%) and the slight rise in pro-inflammatory neutrophils (6.72% *vs*. 5.33%) further indicated an immune imbalance, potentially contributing to the exacerbated inflammation in sepsis.

### 3.3 Functional and dynamic analysis of neutrophils

**Figure 4:**
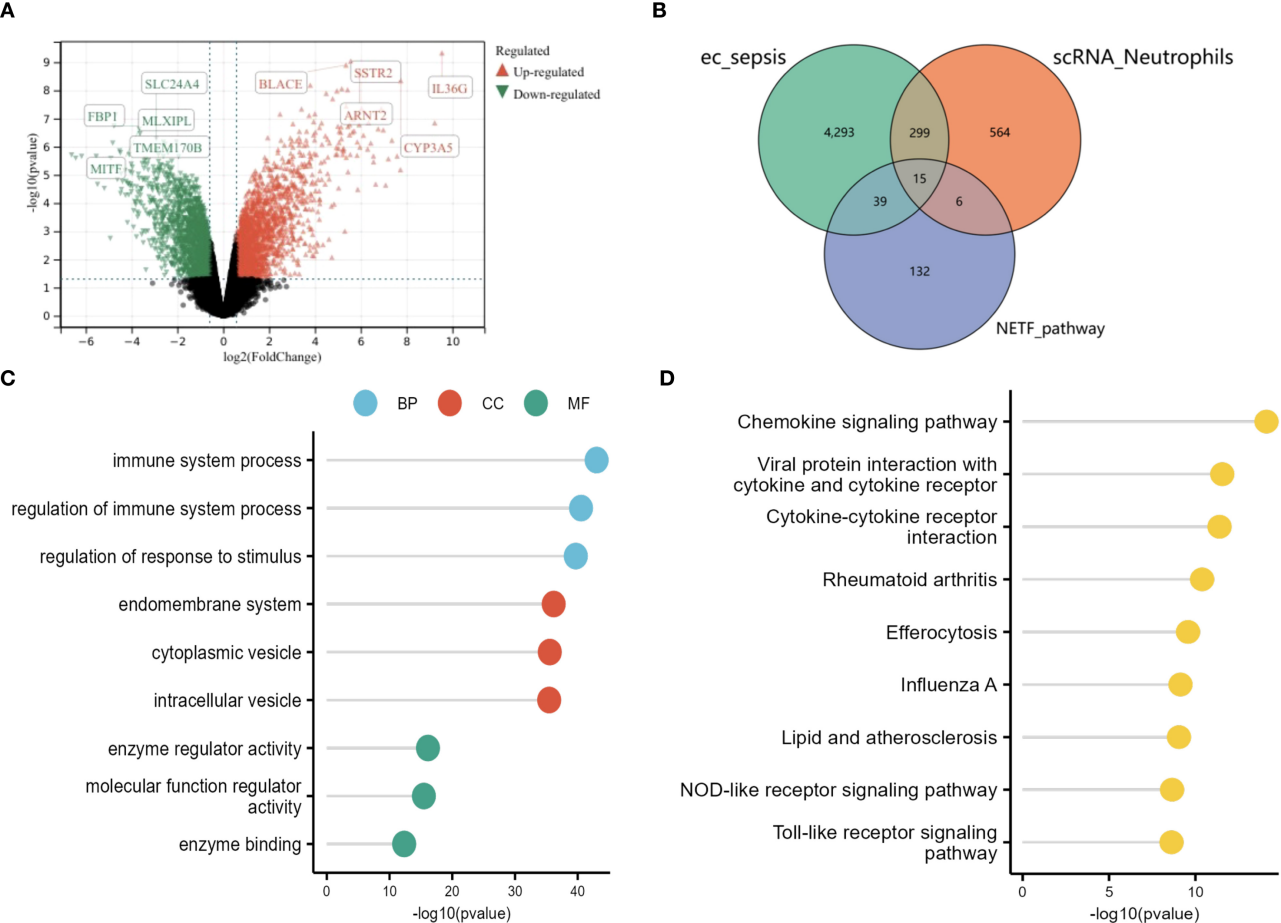
Differential gene expression in sepsis neutrophils. **(A)** Volcano plot of DEGs (GSE237960). **(B)** Venn diagram showing 15 shared genes among neutrophil DEGs, NETF, and **(E)** coli sepsis DEGs. **(C)** GO lollipop plot of top terms. **(D)** KEGG lollipop plot of enriched pathways.

**Figure 5:**
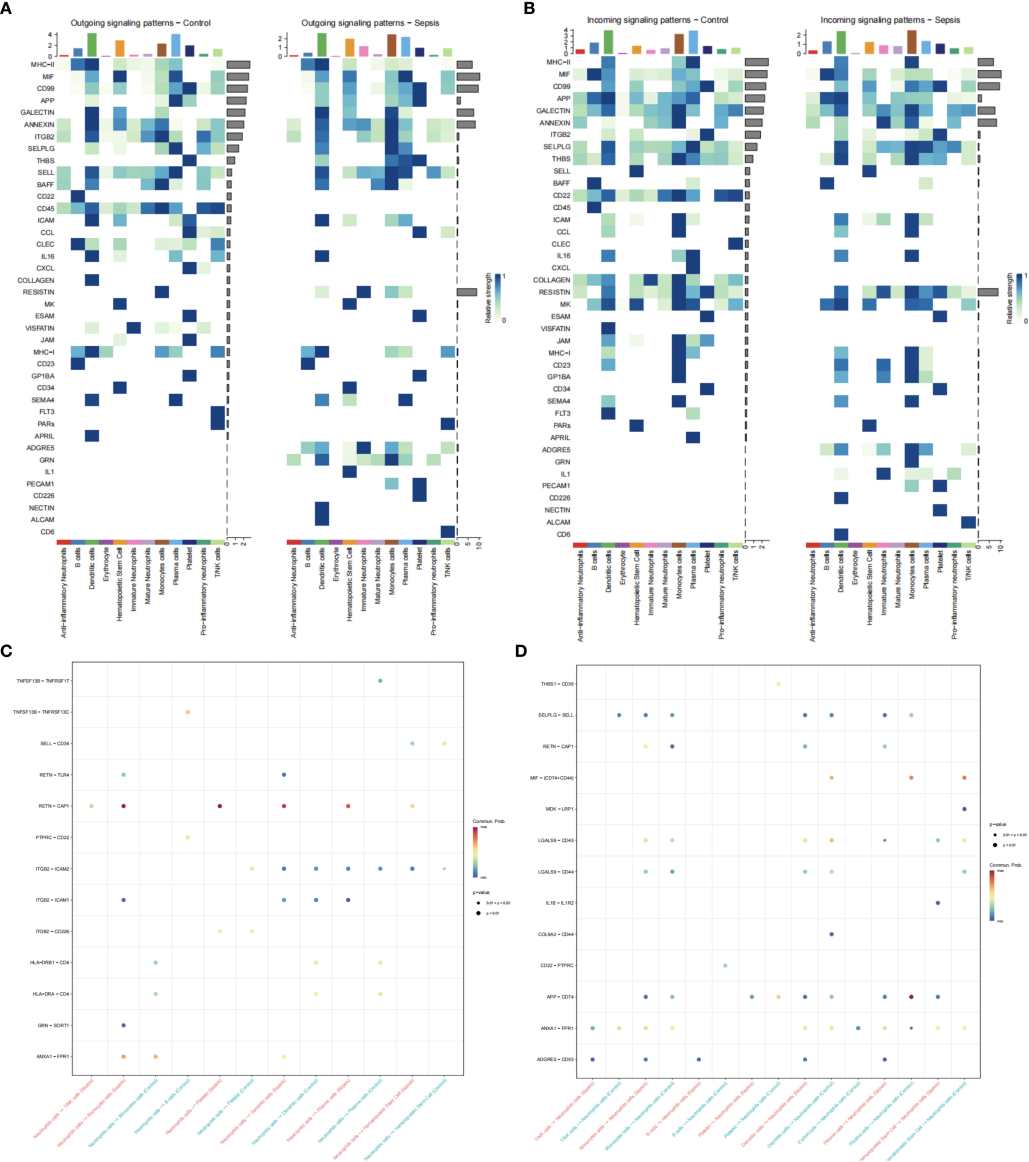
Neutrophil cell communication in sepsis. **(A)** Ligand expression patterns in control *vs*. sepsis. **(B)** Receptor expression patterns. **(C)** Neutrophil ligand interactions. **(D)** Neutrophil receptor interactions.

**Figure 6:**
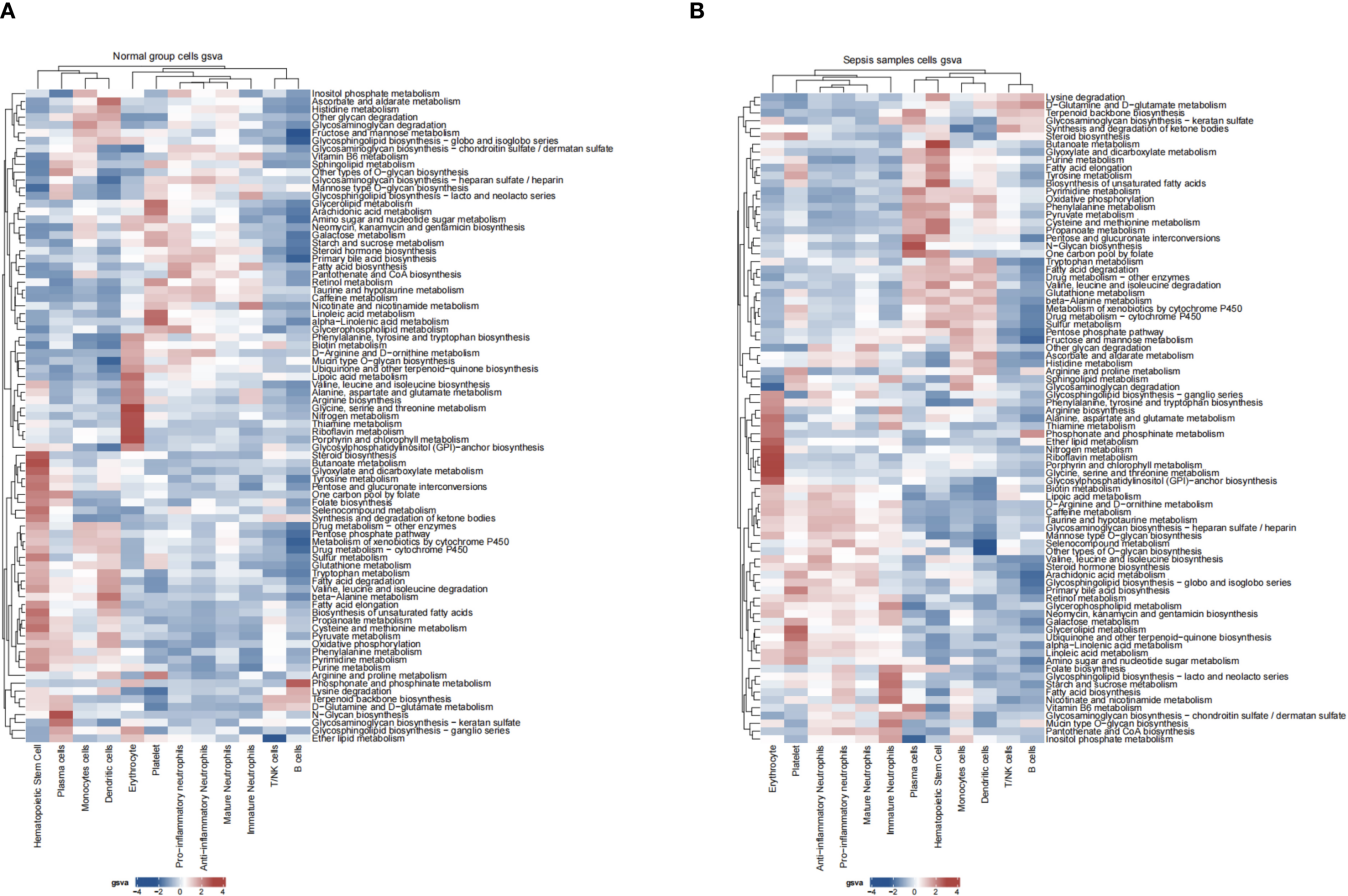
Neutrophil metabolic pathways in sepsis. **(A)** Heatmap of metabolic pathway activity in control group. **(B)** Heatmap in sepsis group.

**Figure 7:**
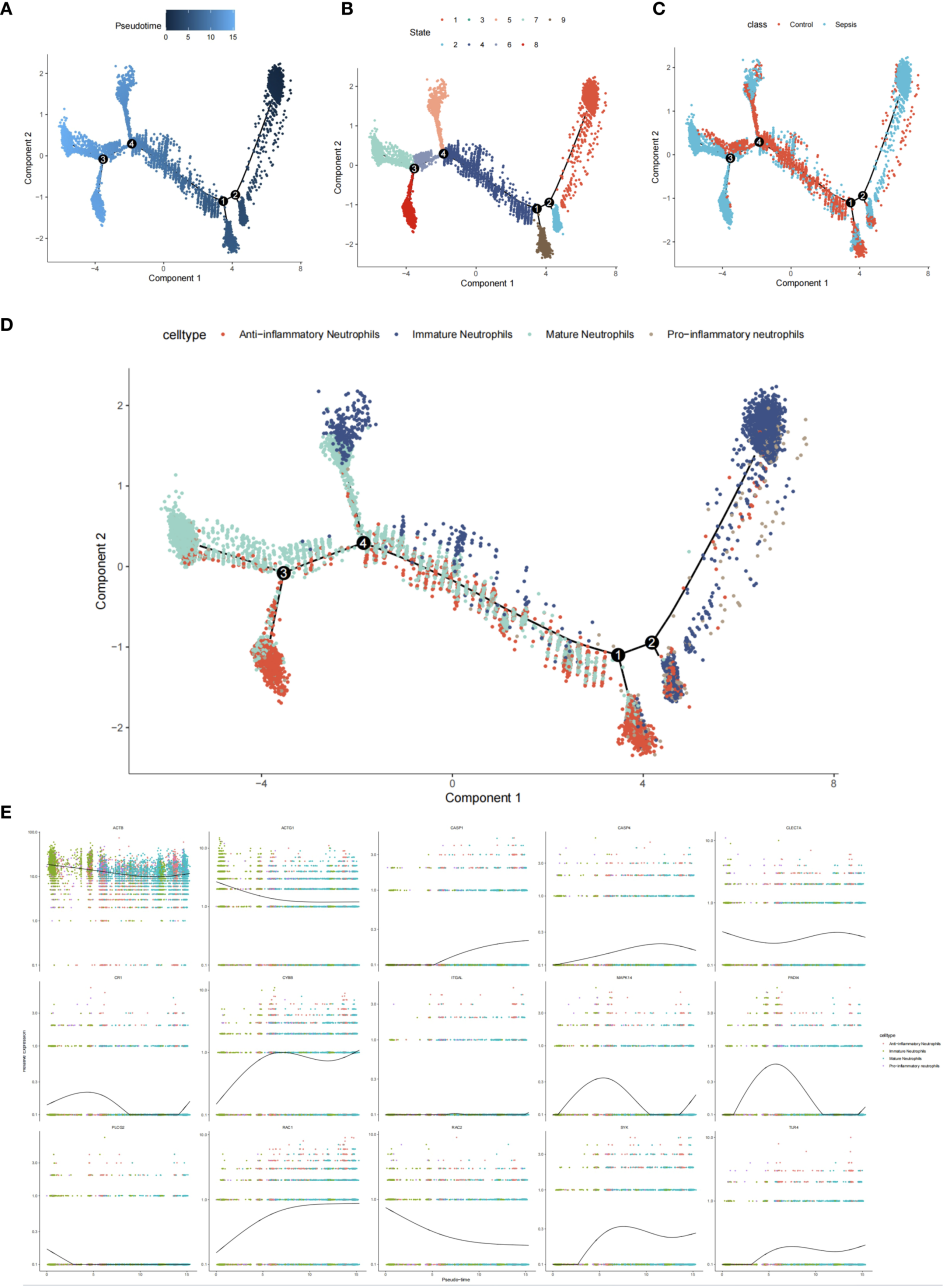
Neutrophil pseudotime analysis in sepsis. **(A)** Pseudotime trajectory plot. **(B)** Pseudotime state distribution. **(C)** Group-specific pseudotime (control *vs*. sepsis). **(D)** Subtype distribution along pseudotime. **(E)** Expression of 15 hub genes.

**Figure 8:**
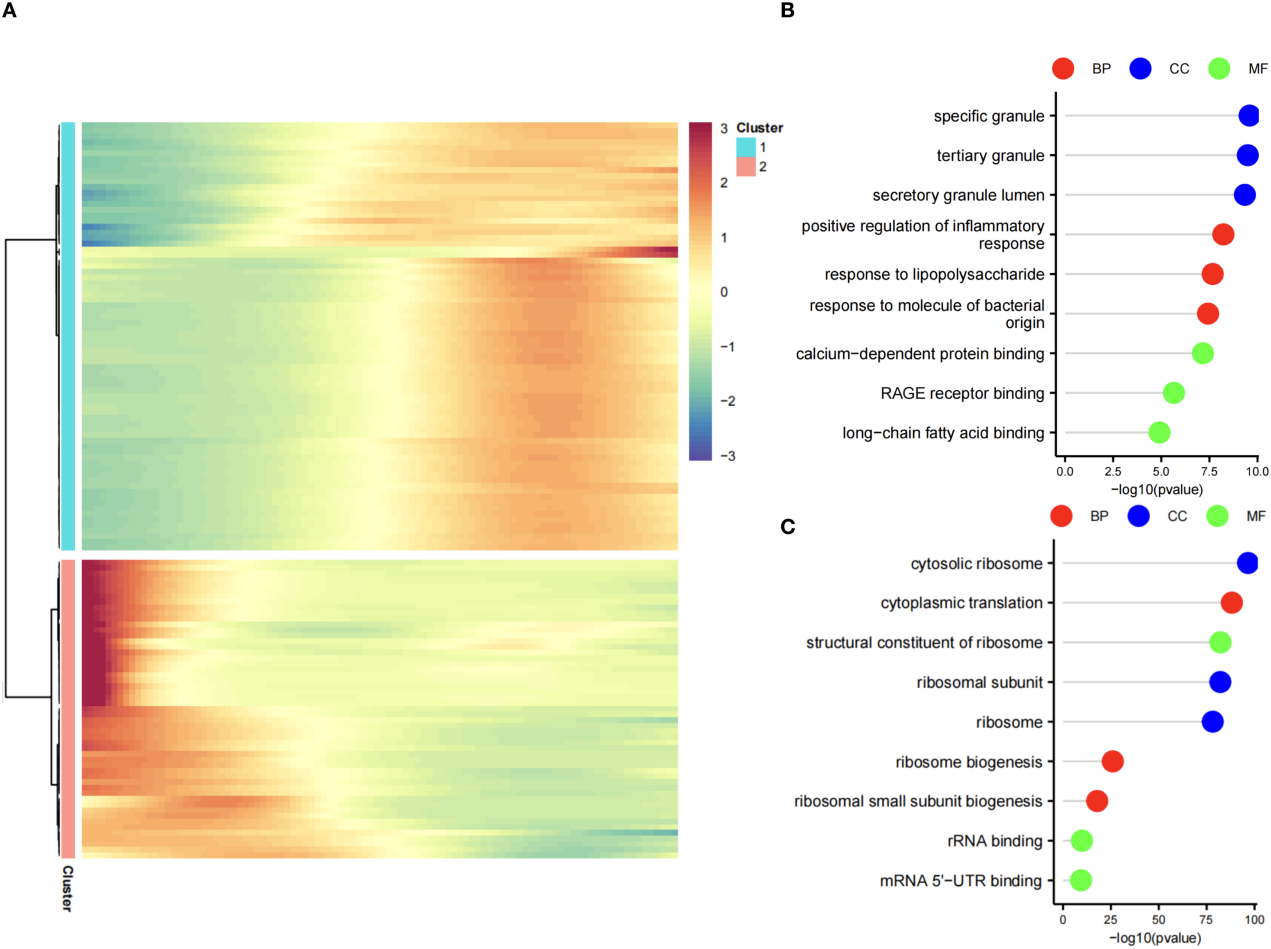
Gene clustering in neutrophil pseudotime. **(A)** Heatmap of two gene clusters: Cluster 1 (early low, late high), Cluster 2 (early high, late low). **(B)** GO lollipop plot for Cluster 1 (immune processes). **(C)** GO lollipop plot for Cluster 2 (protein synthesis).

Differential expression analysis (GSE237960, Escherichia coli-induced sepsis) identified 4646 differentially expressed genes (DEGs), with 2312 upregulated and 2334 downregulated (**Figure 4A**). To narrow down key neutrophil-specific genes, DEGs were intersected across neutrophil pseudotime, neutrophil extracellular trap formation (NETF) pathways, and E. coli-induced sepsis, resulting in 15 shared genes (**Figure 4B**). These DEGs were enriched in chemokine signaling, cytokine-cytokine receptor interaction, and Toll-like receptor pathways (**Figures 4C, D**), reflecting enhanced immune and inflammatory responses in sepsis. Cell communication analysis revealed increased IL-1 signaling (e.g., IL1A-FPR1, IL1B-FPR1) in the sepsis group, suggesting a stronger pro-inflammatory response and potential therapeutic relevance of IL-1 receptor antagonists (e.g., Anakinra). Suppression of HLA-DRB1-CD4 signaling (**Figures 5A–D**) indicated impaired antigen presentation, which may contribute to adaptive immune dysregulation. In the control group, metabolic activities such as glycolysis and fatty acid metabolism were predominant in mature neutrophils (**Figures 6A, B**). In the sepsis group, we observed a significant upregulation of biotin and lipoic acid metabolism in mature neutrophils (**Figure 6B**)—a pattern also observed in immature and pro-inflammatory subtypes—suggesting metabolic reprogramming under inflammatory conditions. Pseudotime analysis revealed a continuous trajectory of neutrophil states (**Figure 7A**), with distinct state distributions reflecting developmental progression (**Figure 7B**). A shift toward an early pro-inflammatory state was observed in sepsis neutrophils compared to controls (**Figure 7C**), with immature subtypes enriched in early stages and mature and anti-inflammatory subtypes in later stages (**Figure 7D**). The expression patterns of the 15 shared genes along the pseudotime axis further highlighted their dynamic regulation, with genes like PADI4 showing elevated expression in early pro-inflammatory stages (**Figure 7E**). Gene clustering revealed two expression patterns: Cluster 1 (low early, high late) enriched in immune processes, and Cluster 2 (high early, low late) enriched in protein synthesis (**Figures 8A–C**), illustrating dynamic neutrophil regulation in sepsis.

### 3.4 Hub gene identification

**Figure 9:**
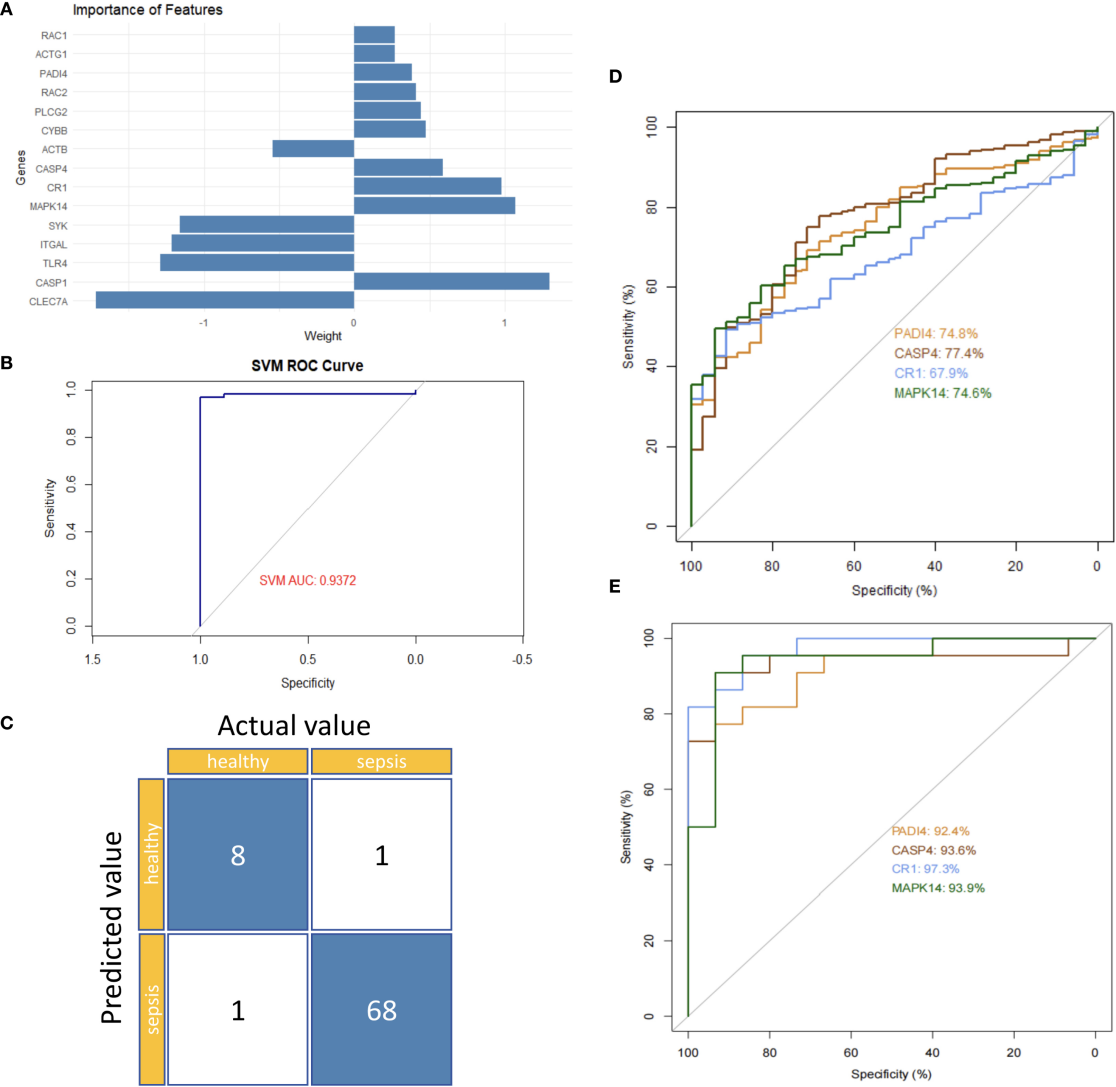
Hub gene validation in sepsis. **(A)** Feature importance ranking of 15 genes. **(B)** Internal ROC curve (GSE185263). **(C)** Confusion matrix (GSE185263). **(D)** Single-gene ROC curves (GSE185263). **(E)** Single-gene ROC curves (GSE243217).

To identify hub genes linked to neutrophil function in sepsis, differential gene expression data were integrated and validated externally using a support vector machine (SVM) model. An SVM model trained on 70% of the GSE185263 dataset identified PADI4, CASP4, CR1, and MAPK14 as significant feature genes (**Figure 9A**). Internal validation yielded an AUC of 0.9372, with only 2 misclassified samples (**Figures 9B, C**). External validation using GSE243217 confirmed high AUC values: PADI4 (92.4%), CASP4 (90.6%), CR1 (97.3%), and MAPK14 (93.9%) (**Figures 9D, E**). The consistent differential expression and high AUC values across datasets underscored their reliability as hub genes in sepsis.

### 3.5 Functional analysis and therapeutic potential of hub genes

**Figure 10:**
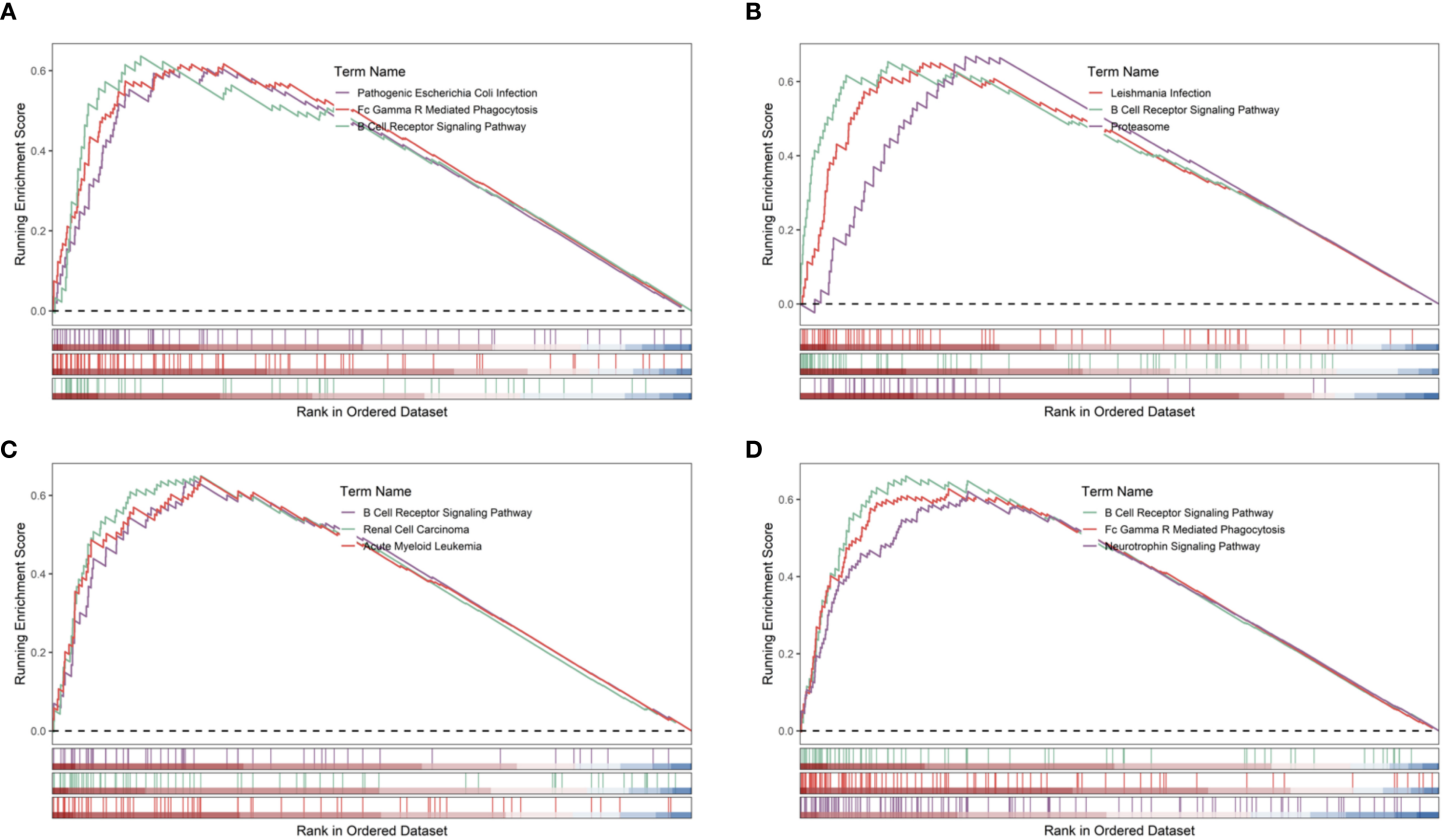
GSEA of hub genes in sepsis. **(A–D)** GSEA plots for PADI4, CASP4, CR1, MAPK14 (GSE185263).

**Figure 11:**
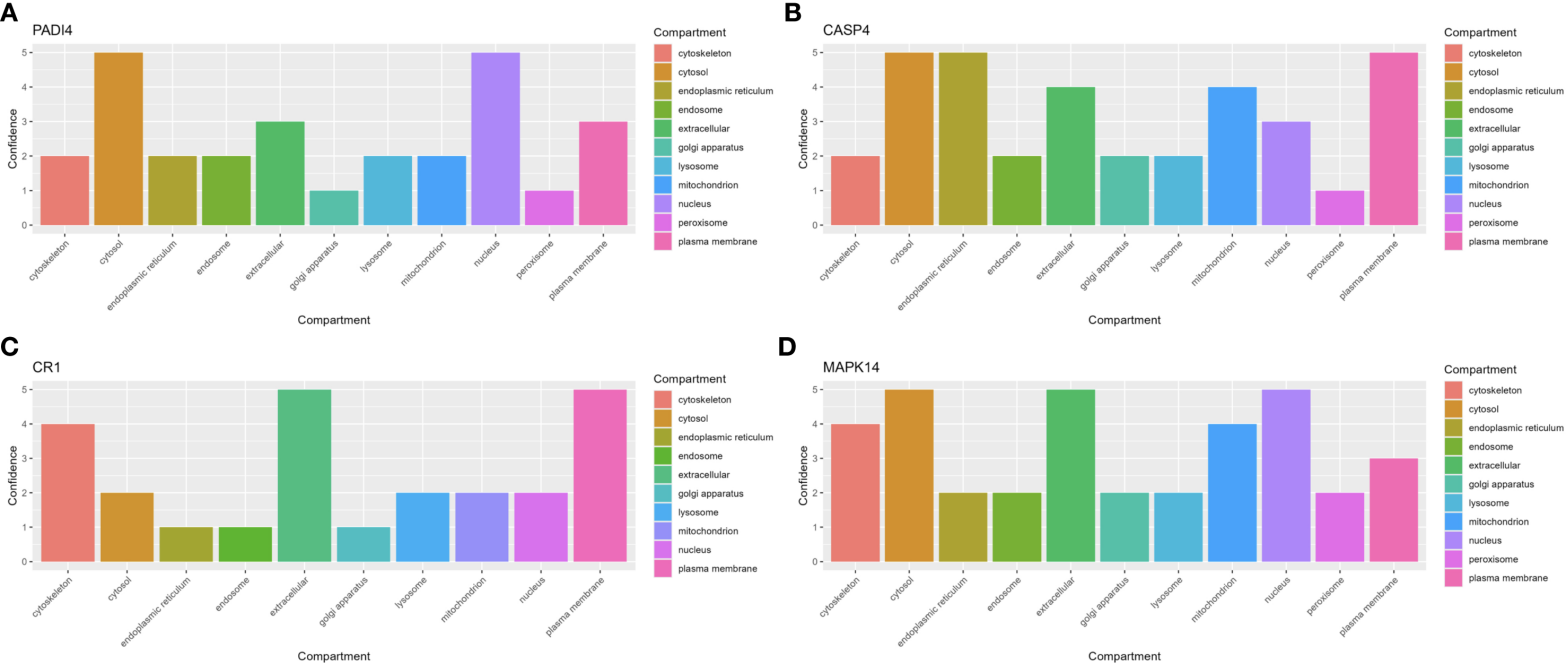
Subcellular localization of hub genes. **(A–D)** Bar plots of PADI4, CASP4, CR1, MAPK14 localization.

**Figure 12:**
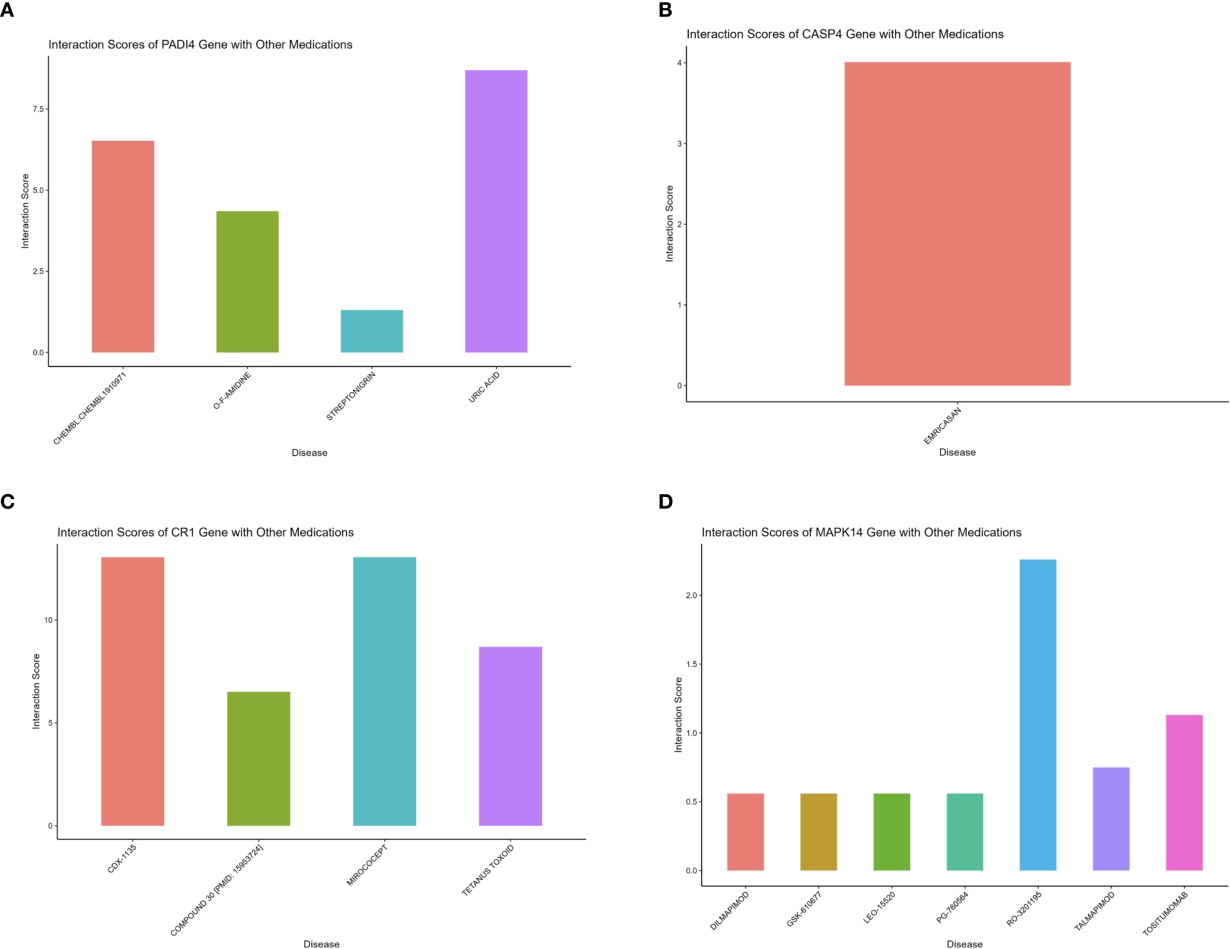
Drug-gene interactions for sepsis therapy. **(A–D)** Bar plots of drug interactions with PADI4, CASP4, CR1, MAPK14.

Gene set enrichment analysis (GSEA) indicated that all four hub genes were enriched in immune and inflammation-related pathways, suggesting synergistic roles in sepsis immune regulation. Specifically, PADI4 was enriched in Pathogenic Escherichia coli Infection (NES = 1.62, p = 8.56e-07, FDR = 1.22e-05) and Fc Gamma R-Mediated Phagocytosis (NES = 1.62, p = 9.31e-10, FDR = 1.47e-07, **Figure 10A**). CASP4 showed enrichment in Leishmania Infection (NES = 1.70, p = 1.03e-08, FDR = 2.80e-07), B Cell Receptor Signaling Pathway (NES = 1.69, p = 1.04e-09, FDR = 3.88e-08), and Proteasome (NES = 1.67, p = 1.10e-06, FDR = 1.14e-05, **Figure 10B**). CR1 and MAPK14 were enriched in B Cell Receptor Signaling and Renal Cell Carcinoma pathways (**Figures 10C, D**). Subcellular localization analysis revealed PADI4 primarily in the cytosol and nucleus, CASP4 in the endoplasmic reticulum and cytosol, CR1 in the extracellular space and plasma membrane, and MAPK14 in the cytosol, extracellular space, and nucleus (**Figures 11A–D**), supporting their functional diversity. Drug-gene interaction analysis suggested potential therapeutic candidates, including uric acid for PADI4, emricasan for CASP4, and RO-3201195 for MAPK14 (**Figures 12A–D**), offering directions for targeted sepsis therapies. Consistent with this prediction, preclinical studies demonstrated that febuxostat treatment lowered tissue urate levels and significantly reduced citH3 expression in mice (34), indicating suppression of PAD4-dependent NETosis *in vivo*.

### 3.6 Immune microenvironment and clustering analysis

**Figure 13:**
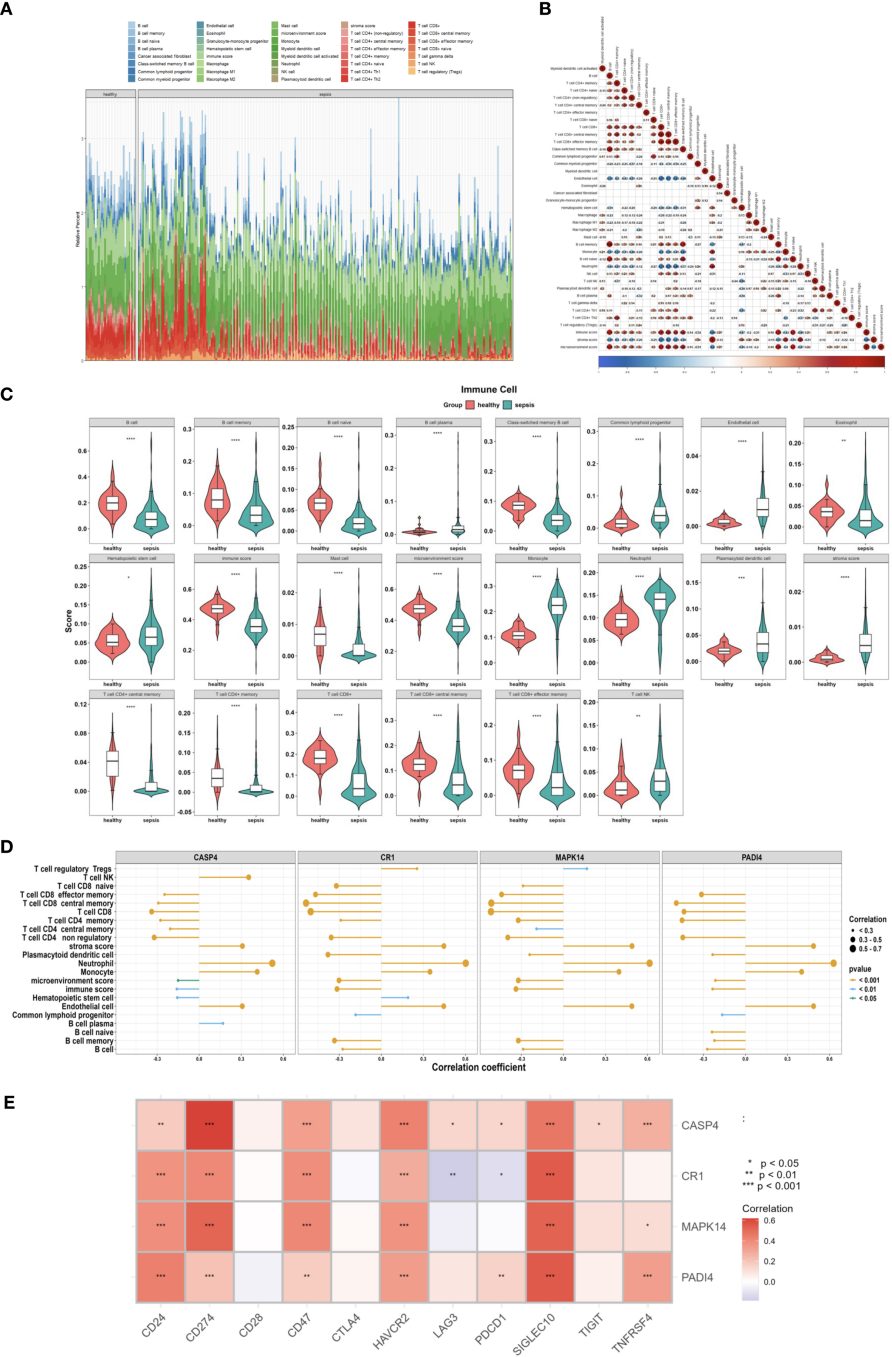
Immune microenvironment in sepsis. **(A)** Stacked bar plot of 36 immune cell proportions (GSE185263). **(B)** Correlation heatmap of immune cells. **(C)** Violin plot of differentially enriched cells. **(D)** Lollipop plot of gene-immune cell correlations. **(E)** Heatmap of immune checkpoint gene correlations.

**Figure 14:**
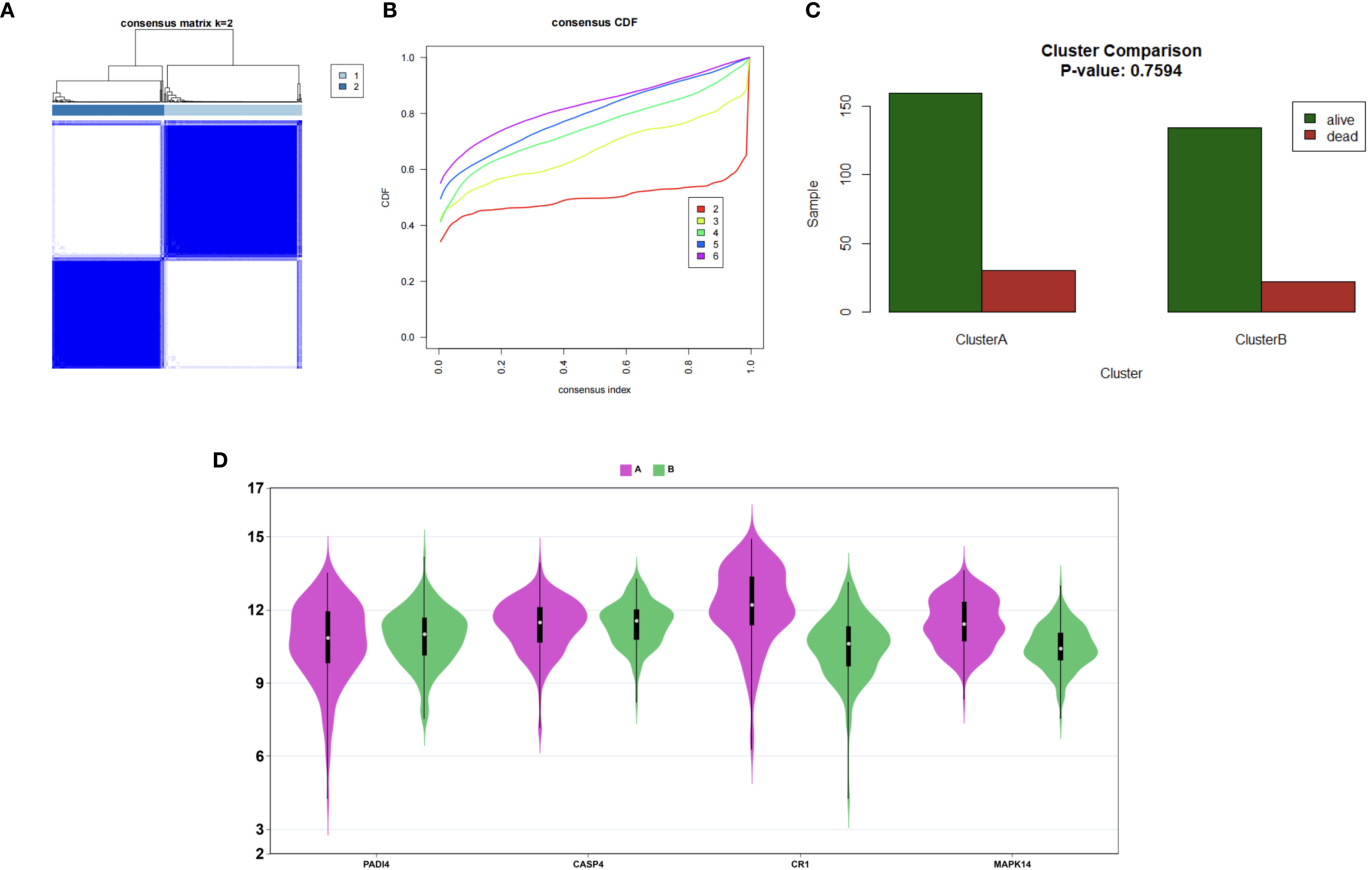
Patient clustering in sepsis. **(A)** Consensus matrix (k=2). **(B)** CDF plot for cluster numbers. **(C)** Survival bar plot of Clusters A and B. **(D)** Violin plots of hub gene expression.

Immune microenvironment analysis revealed significantly increased neutrophil infiltration (P<0.001) and reduced T cell infiltration (e.g., CD4+ memory T cells, P<0.001) in the sepsis group (**Figures 13A, C**), with a strong negative correlation between neutrophils and T cells (P<0.001, **Figure 13B**). PADI4, CASP4, CR1, and MAPK14 were positively correlated with neutrophil infiltration (P<0.001) and negatively correlated with T cell infiltration (P<0.001, **Figure 13D**), and positively associated with immune checkpoint genes (CD24, CD47, P<0.01, **Figure 13E**), suggesting immunosuppressive mechanisms. The increased neutrophil infiltration and decreased T cell presence indicated an imbalance between innate and adaptive immunity in sepsis, potentially linked to disease severity. To corroborate these xCell-derived profiles, CIBERSORT analysis was applied to the same bulk RNA-seq data, estimating proportions of 22 immune cell types. Consistent with xCell findings, it confirmed significantly elevated neutrophil fractions in the sepsis group (P < 0.001; **Supplementary Figures 1A, C**) and reduced T cell subtypes, such as CD4 memory activated T cells . Neutrophils and T cells showed a strong negative correlation (**Supplementary Figure 1B**), while the hub genes exhibited positive correlations with neutrophil infiltration (r = 0.3–0.7, **Supplementary Figure 1C**). These genes were also positively associated with immune checkpoint molecules (**Supplementary Figure 1E**), reinforcing the immunosuppressive microenvironment. Consensus clustering divided patients into two clusters: Cluster A exhibited lower PADI4 and CASP4 expression and higher CR1 and MAPK14 expression (**Figures 14A–D**), highlighting molecular heterogeneity. However, survival differences between clusters were not statistically significant (p=0.7594), possibly due to limited sample size, necessitating further clinical validation.

### 3.7 Validation of hub genes in sepsis

**Figure 15:**
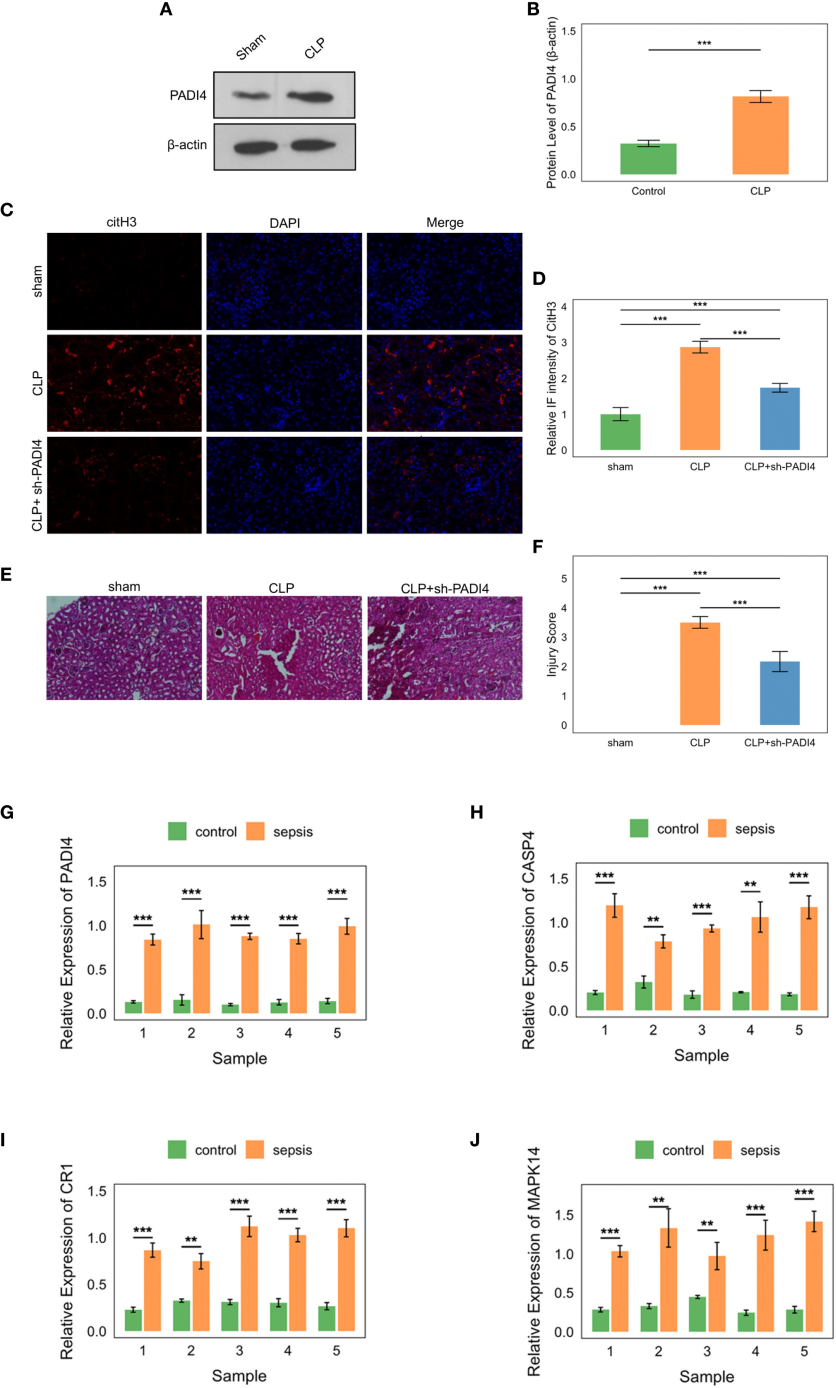
Experimental validation of hub genes in sepsis. **(A, B)** Western blot showing PADI4 upregulation in CLP model kidney tissues (P<0.001). **(C, D)** Immunofluorescence of citH3, reduced by PADI4 knockdown (P<0.001). **(E, F)** H&E staining showing attenuated AKI pathology post-PADI4 knockdown (P<0.001). **(G-J)** qRT-PCR confirming elevated PADI4, CASP4, CR1, MAPK14 in sepsis patient blood (P<0.05).

To investigate the role of hub genes in sepsis-associated kidney injury, we employed a cecal ligation and puncture (CLP) model to induce sepsis in mice, followed by extraction of kidney tissues for *in vitro* experimental analyses to assess the expression differences of PADI4. Western blot (WB) analysis revealed a significant upregulation of PADI4 expression in the kidney tissues of the CLP group compared to the sham-operated group, with quantitative analysis (normalized to β-actin) confirming a markedly higher relative expression level (p < 0.001, **Figures 15A, B**). The full uncropped Western Blot images are provided in **Supplementary Figure S2**. Immunofluorescence (IF) staining further demonstrated a pronounced increase in the red fluorescence signal of citrullinated histone H3 (citH3, a marker of neutrophil extracellular trap [NET] formation) in the kidney tissues of the CLP group, significantly exceeding that of the sham group (p < 0.001). Notably, knockdown of PADI4 resulted in reduced citH3 expression, suggesting a critical role for PADI4 in sepsis-induced NET formation (p < 0.001 compared to the CLP group, **Figures 15C, D**). Hematoxylin and eosin (H&E) staining revealed significant pathological changes in the kidney tissues of the CLP group, characterized by tubular dilation, expansion of the tubulointerstitial area, and inflammatory cell infiltration, consistent with the features of sepsis-induced acute kidney injury. The injury score was significantly higher in the CLP group than in the sham group (p < 0.001). However, PADI4 knockdown markedly attenuated these pathological changes (p < 0.01 compared to the CLP group, **Figures 15E, F**), indicating that PADI4 silencing significantly ameliorates sepsis-induced renal histopathological damage. Furthermore, we validated the elevated expression of the hub genes in the peripheral blood of patients with sepsis, consistent with our previous findings (compared to the control group, **Figures 15G–J**).

## 4 Discussion

In this study, we identified four neutrophil subtypes, with the delineation of pro-inflammatory and anti-inflammatory subtypes representing a significant innovation. Pro-inflammatory neutrophils were characterized by genes such as PF4, CXCR2, CXCL8, and CCL5, which play critical roles in amplifying inflammation. These genes enhance inflammatory responses through chemotaxis, degranulation, and reactive oxygen species production (29, 35). For instance, CXCL8 mediates a positive feedback loop via CXCR2, promoting neutrophil recruitment (32). This self-amplifying signaling pathway is particularly prominent in chronic lung diseases such as chronic obstructive pulmonary disease and bronchiectasis (36). As a chemokine, CCL5 effectively attracts neutrophils to sites of infection or injury, thereby augmenting immune and inflammatory responses (30, 31). During the acute phase of sepsis, Ccl5-positive macrophages exhibited the strongest interaction with neutrophils, predominantly through the CCL signaling pathway, suggesting that Ccl5-positive macrophages play a key role in neutrophil recruitment via CCL5-CCR1 pairing (33)。In contrast, anti-inflammatory neutrophils were distinguished by high expression of MT-ND1, MT-CO1, MALAT1, and CST3, which are associated with mitochondrial function, inflammation resolution, and tissue protection. MT-ND1 and MT-CO1, core components of the mitochondrial electron transport chain (complexes I and IV, respectively), maintain mitochondrial membrane potential and energy metabolism balance, supporting normal neutrophil function. Mitochondria contribute to inflammation resolution by regulating neutrophil apoptosis, exerting an anti-inflammatory effect (27). Studies have shown that inhibitors of complexes I and III, such as rotenone and antimycin A, significantly reduce neutrophil migration (26). The anti-inflammatory effect of MALAT1 is primarily mediated by its regulation of IL-10 expression. In a Leishmania Donovani model, MALAT1 knockout mice exhibited reduced IL-10 expression in CD4-positive T cells, enhancing macrophage activation and pathogen clearance (28). CST3 exerts its effects mainly by inhibiting cysteine proteases, with studies demonstrating that high cystatin C concentrations significantly suppress spontaneous, fMLP-, and PMA-induced neutrophil respiratory bursts (37). In the sepsis group, the proportions of pro-inflammatory and immature neutrophils were significantly elevated (40.53% and 6.72% *vs*. 4.19% and 5.33% in controls), while anti-inflammatory neutrophils decreased (18.43% *vs*. 27.04%), consistent with a shift toward an early pro-inflammatory state in pseudotime analysis. This suggests that neutrophils in the acute phase of sepsis predominantly adopt an early pro-inflammatory state, reflecting immune mobilization against infection. However, this imbalance may lead to uncontrolled inflammation, exacerbating damage to renal tubular epithelial and endothelial cells, which aligns with the high incidence of sepsis-associated acute kidney injury. These findings not only elucidate the role of neutrophil heterogeneity in immune dysregulation during urosepsis but also provide a foundation for developing strategies to suppress pro-inflammatory subtypes or enhance the reparative functions of anti-inflammatory subtypes.

Beyond phenotypic heterogeneity, the dynamic regulation of neutrophil function is increasingly recognized as a critical determinant of sepsis outcomes. Recent studies have demonstrated that metabolic reprogramming and inflammatory signaling shape neutrophil effector functions, such as NET formation and cytokine release, thereby amplifying tissue injury in sepsis. Although direct mechanistic evidence in sepsis-associated AKI remains limited, related bioinformatics and network-based studies from other immune-inflammatory conditions have highlighted the interplay between metabolic pathways and immune cell heterogeneity (38–40). While these studies are not focused on sepsis per se, they provide methodological and contextual insights supporting the concept that neutrophil subtypes may adopt distinct functional states under systemic inflammatory stress. Our findings extend this perspective by integrating multi-omics data with experimental validation, specifically linking PAD4-driven pro-inflammatory neutrophils to renal injury in sepsis.

Integrative analysis of single-cell and bulk RNA sequencing data, combined with machine learning, further identified PADI4, CASP4, CR1, and MAPK14 as hub genes in sepsis. These genes exhibited high expression in neutrophils and positively correlated with neutrophil infiltration, underscoring their significance in the inflammatory response during sepsis. Neutrophil extracellular traps (NETs) have emerged as important players in sepsis. Although extracellular in nature, their components can directly injure renal tubular cells (41). Notably, urosepsis may differ immunologically from other sepsis types, yet NETs remain scarcely studied in this context. To address this gap, we focused on NETs-related genes and intersected them with E. coli-induced and neutrophil-associated gene sets. PADI4 plays a dual role in sepsis by mediating NETosis (neutrophil extracellular trap formation). On one hand, it contributes to infection defense by capturing pathogens such as Escherichia coli through NETs, a process involving the conversion of arginine residues in histones to citrulline, significantly reducing histone positive charge (42, 43). This modification weakens the electrostatic interactions between positively charged histones and negatively charged DNA, leading to nucleosome destabilization and chromatin decondensation (44). Studies have shown that in PADI4-deficient neutrophils, histone H3 citrullination is completely absent, and NETosis induced by PMA and LPS does not occur (45). Moreover, PADI4 inhibitors significantly block histone citrullination and NET release (46, 47). On the other hand, excessive NET release may exacerbate renal tissue damage. Gene set enrichment analysis revealed PADI4 enrichment in the Escherichia coli infection pathway (NES = 1.62, FDR = 1.22e-05), supporting its specific role in sepsis. The therapeutic implications of targeting PADI4 merit further consideration. Preclinical evidence supports that pharmacological inhibition of PADI4 with small-molecule inhibitors, such as Cl-amidine (a pan-PAD inhibitor), significantly reduces renal ischemia/reperfusion-induced acute kidney injury, attenuates tubular inflammation, and decreases neutrophil infiltration in murine models (48, 49). Furthermore, specific PAD4 inhibition using GSK484 has demonstrated renal protection by reducing remote lung injury, NET formation, and histone H3 citrullination in acute kidney injury contexts (50). Although these inhibitors are not yet in clinical use, they provide a compelling proof-of-concept that PADI4 inhibition holds promise as a therapeutic strategy for SAKI. CASP4 directly binds cytoplasmic lipopolysaccharide, particularly its active component lipid A, via its CARD domain, forming a multimeric complex that activates its enzymatic activity and induces pyroptosis (51). This process also triggers NLRP3 inflammasome assembly, promoting the release of IL-1β and IL-18, thereby amplifying the immune response (52)。CR1 facilitates the clearance of immune complexes by binding C3b and C4b (53), preventing their deposition in vulnerable tissues such as the kidney and lungs (54), thus exhibiting anti-inflammatory and renoprotective potential. In systemic lupus erythematosus, studies have reported significantly reduced CR1 expression on erythrocytes and leukocytes, correlating with disease severity and renal involvement (55). MAPK14 is activated in response to various stimuli, including cytokines (e.g., TNF-α, IL-1β), oxidative stress, lipopolysaccharide, and viral infections (56). MAPK14 activates the downstream kinase MK2, which phosphorylates the RNA-binding protein TTP, preventing degradation of AU-rich element-containing mRNAs and stabilizing pro-inflammatory cytokine mRNAs such as TNF-α, IL-1β, and IL-6 (56, 57). This mechanism is particularly pronounced in macrophages and dendritic cells, promoting rapid inflammatory responses. MAPK14 also directly phosphorylates transcription factors such as NF-κB, ATF2, and MEF2, enhancing the transcriptional activity of pro-inflammatory genes (57).

The pseudotime trajectory analysis revealed an early pro-inflammatory state shift in neutrophils during sepsis, characterized by increased expression of pro-inflammatory genes such as PADI4, which aligns with the significant rise in pro-inflammatory neutrophil proportions (40.53% in sepsis *vs*. 4.19% in controls, **Figure 3D**). This suggests that the early pro-inflammatory state may drive the expansion of pro-inflammatory neutrophils, potentially exacerbating renal damage through enhanced NETosis and inflammatory cascades. The concomitant decrease in anti-inflammatory neutrophils (18.43% in sepsis *vs*. 27.04% in controls) could reflect a shift in neutrophil maturation or functional reprogramming along the pseudotime axis, as supported by dynamic gene expression patterns (**Figure 7E**). Previous studies have demonstrated that pseudotime analysis can uncover cell state transitions linked to subtype proportions, and our GSEA results (**Figure 10**) further indicate that pathways such as MAPK signaling (enriched for MAPK14) may underpin this transition.

Analysis of the immune microenvironment revealed significantly increased neutrophil infiltration and decreased T cell infiltration in the sepsis group, reflecting an imbalance between hyperactive innate immunity and suppressed adaptive immunity. This imbalance may exacerbate renal damage through excessive activation of pro-inflammatory neutrophils. The positive correlation between hub genes and immune checkpoint genes (CD24, CD47, HAVCR2; ρ = 0.40-0.80, P < 0.01) suggests an immunosuppressive mechanism. Previous studies have shown that PD-L1 expression on neutrophils is upregulated during sepsis, potentially suppressing adaptive immunity via the PD-L1/PD-1 checkpoint (58), a phenomenon also observed in our study. This T cell suppression, driven by immune checkpoint activation, may have implications for secondary infections, a common complication in sepsis linked to immunoparalysis (59). Literature indicates that T cell dysfunction, particularly through PD-1/PD-L1 signaling (60), impairs pathogen clearance and increases susceptibility to opportunistic infections such as Gram-negative bacteria (61). The observed suppression, potentially influenced by neutrophil-driven inflammation and hub gene activities (e.g., PADI4, MAPK14), could contribute to this risk. CD24 interacts with SIGLEC10 to inhibit macrophage phagocytosis (62). Li et al. suggested that the CD24-SIGLEC10 signaling pathway is a potential target for cancer immunotherapy, as suppressing immune responses may reduce inflammatory damage (63). CD47, a widely expressed transmembrane protein, acts as a “don’t eat me” signal by interacting with SIRPα on macrophages to inhibit phagocytosis. Casey et al. found that MYC regulates CD47 expression, with its high expression suppressing anti-tumor immune responses (64). HAVCR2 (TIM-3), an innate and adaptive immune checkpoint molecule, is associated with T cell exhaustion when highly expressed. Pan-cancer analyses have shown that elevated HAVCR2 expression correlates with immune infiltration and checkpoint genes, potentially serving as a key factor in immunosuppression (65). Based on our findings, we propose that the high expression of these immune checkpoint genes may suppress immune cell activation, reduce inflammation, maintain immune balance, and prevent excessive tissue damage. This mechanism likely contributes to immune homeostasis in the later stages of sepsis. Cell communication analysis indicated enhanced IL-1 signaling and suppressed HLA-DRB1-CD4 signaling, suggesting impaired antigen presentation that may further weaken T cell function. IL-1 receptor antagonists, such as anakinra, may mitigate renal inflammation by blocking this pathway. Metabolic pathway analysis revealed upregulated biotin and lipoic acid metabolism, indicating metabolic reprogramming that supports the pro-inflammatory functions of neutrophils, offering new avenues for metabolic interventions, such as biotin supplementation.

The rat cecal ligation and puncture model and human peripheral blood validation experiments bolstered the reliability of our bioinformatics findings. The cecal ligation and puncture model demonstrated that elevated PADI4 expression (P < 0.001) was associated with enhanced NETosis (increased citH3 signaling), while PADI4 knockdown significantly alleviated acute kidney injury pathology (P < 0.01), supporting PADI4 as a central driver of renal damage by pro-inflammatory neutrophils (45). In human peripheral blood, the high expression of PADI4, CASP4, CR1, and MAPK14 (P < 0.05) was consistent with bulk RNA sequencing results, with ROC analysis (AUC 0.67-0.97) further validating their diagnostic potential.

A notable limitation of this study is the reliance on general sepsis datasets (GSE175453 and GSE167363), which primarily capture systemic immune responses rather than kidney-specific pathology, and the use of the CLP model, which induces systemic sepsis but is not specific to SAKI. The absence of SAKI-specific clinical annotations in the datasets, such as KDIGO-based diagnostic criteria (e.g., serum creatinine or urine output), and the lack of quantitative kidney injury biomarkers (e.g., BUN, CR, NGAL, KIM-1) in the CLP model, limit direct attribution of neutrophil heterogeneity and hub gene effects to SAKI. Instead, our findings elucidate sepsis-induced immune dysregulation, particularly neutrophil-driven mechanisms, that may contribute to organ dysfunction including SAKI. Future studies utilizing SAKI-specific cohorts with detailed clinical data or SAKI-specific models (e.g., urosepsis models) with biomarker quantification could validate and extend these findings to establish direct links with kidney injury.

Despite significant progress, this study has limitations. Public datasets (GSE175453, GSE167363) and the cecal ligation and puncture model primarily reflect general sepsis mechanisms, lacking urosepsis-specific data and models, which limits direct insights into urinary tract infection-induced inflammation. The small sample size of human peripheral blood (n=5) may affect statistical robustness. Experimental validation focused on PADI4, and the roles of other genes, such as CASP4, require further exploration.

For requirements for a specific article type please refer to the Article Types on any Frontiers journal page. Please also refer to Author Guidelines for further information on how to organize your manuscript in the required sections or their equivalents for your field.

## Data Availability

The original contributions presented in the study are included in the article/**Supplementary Material**. Further inquiries can be directed to the corresponding author.
