# Supplementary figures and images for: Multi-omics analysis reveals neutrophil heterogeneity and key molecular drivers in sepsis-associated acute kidney injury
Source: Front Immunol. 2025 Oct 2;16:1637692. doi: 10.3389/fimmu.2025.1637692 (PMC12528136; doi:10.3389/fimmu.2025.1637692)

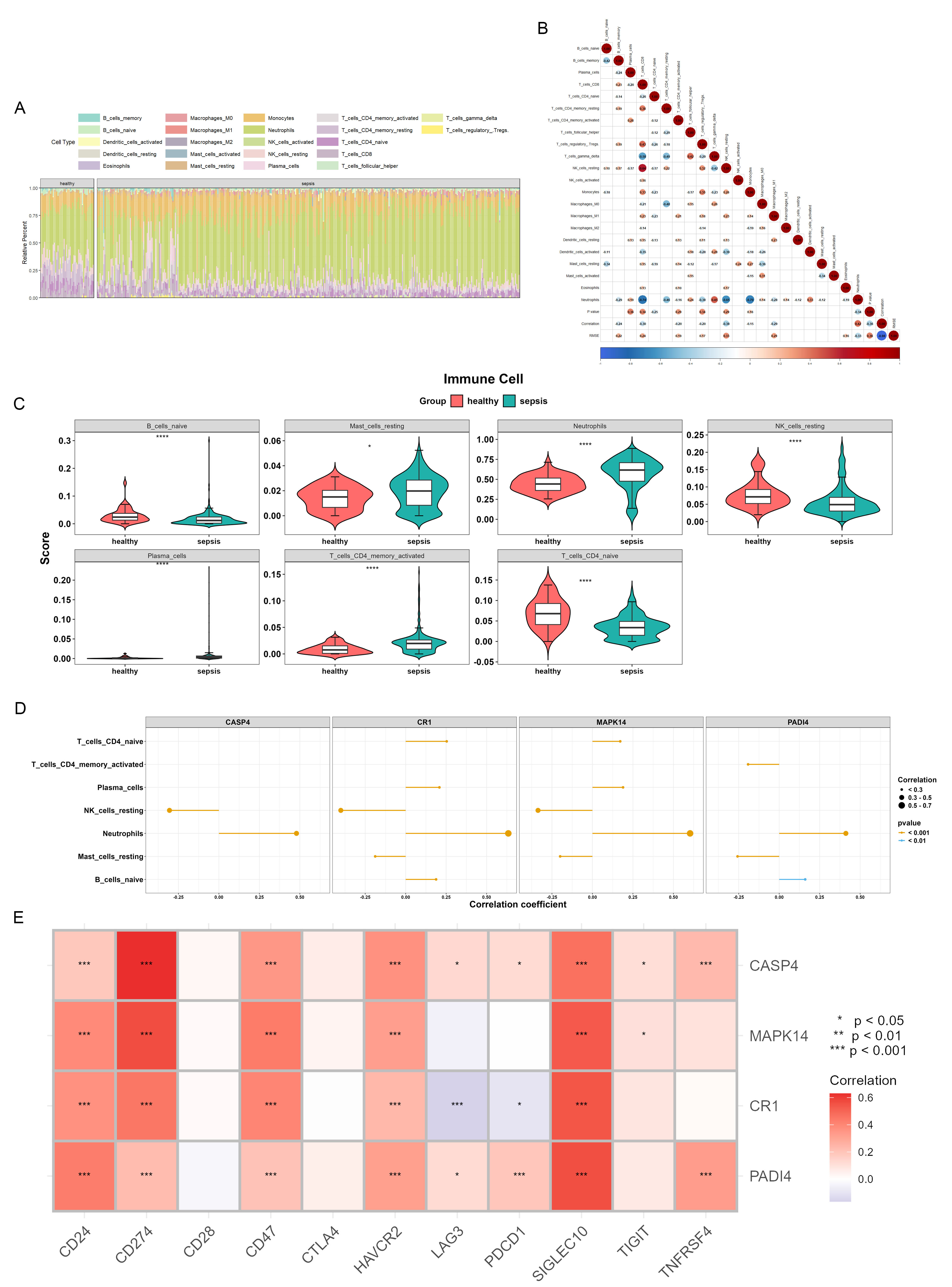

Supplement: Supplementary file 1 [file Image1.jpg]

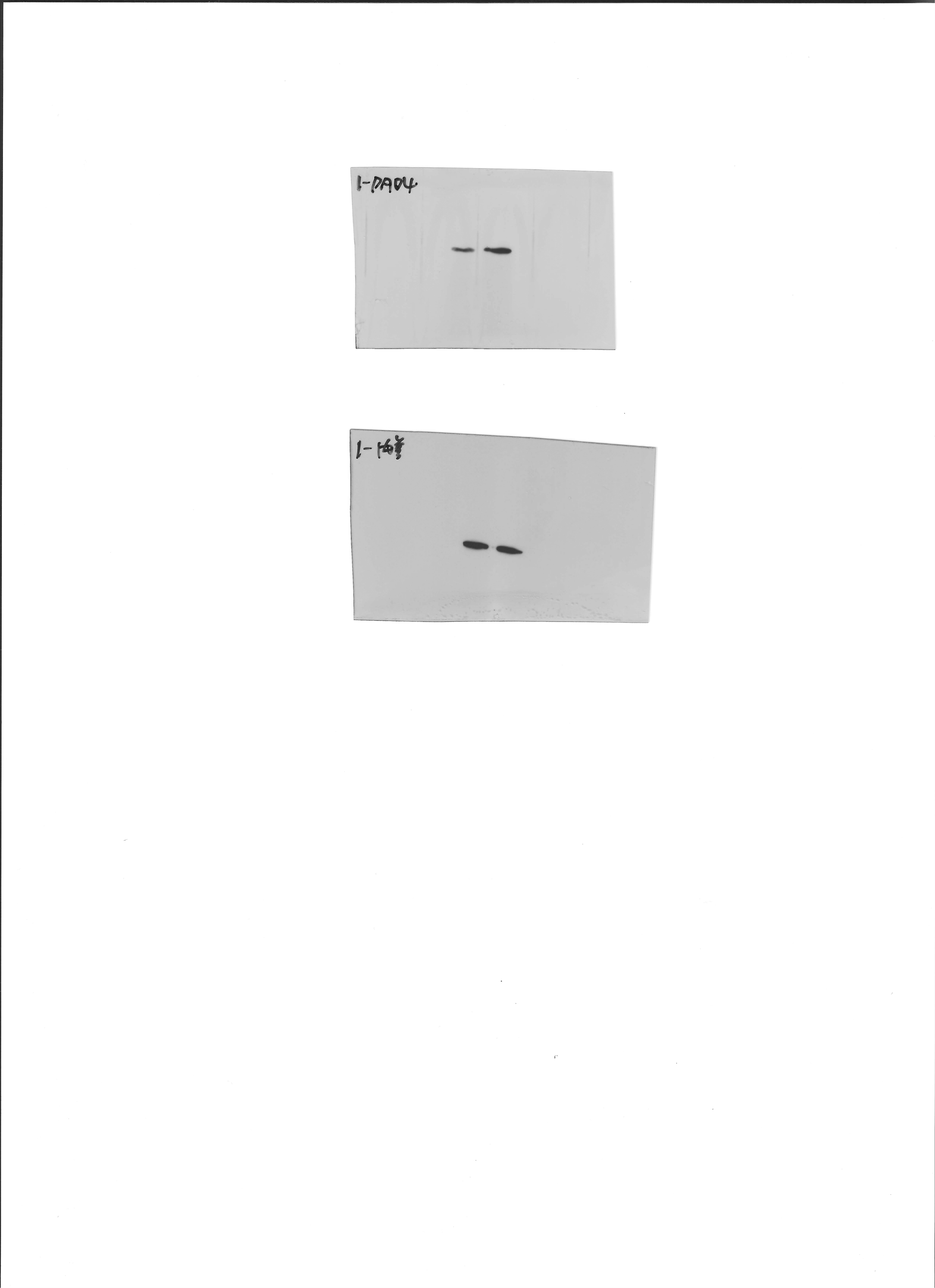

Supplement: Supplementary file 2 [file Image2.jpg]
